# Supplementary material for: Changes in neonatal admissions, care processes and outcomes in England and Wales during the COVID-19 pandemic: a whole population cohort study
Source: BMJ Open. 2021 Oct 1;11(10):e054410. doi: 10.1136/bmjopen-2021-054410 (PMC8488283; doi:10.1136/bmjopen-2021-054410)
Supplement: Supplementary data [file bmjopen-2021-054410supp001.pdf]

**Supplementary Table S1**

UK Neonatal Collaborative hospitals and lead clinicians in England and Wales

| **Hospital** | **Lead clinician** |
|---|---|
| Airedale General | Dr Matthew Babirecki |
| Arrowe Park | Dr Anand Kamalanathan |
| Barnet | Dr Tim Wickham |
| Barnsley District General | Dr Kavi Aucharaz |
| Basildon | Dr Aashish Gupta |
| Basingstoke & North Hampshire | Dr Nicola Paul |
| Bassetlaw District General | Dr L M Wong |
| Bedford | Dr Anita Mittal |
| Birmingham City | Dr Penny Broggio |
| Birmingham Heartlands | Dr Pinki Surana |
| Birmingham Women's | Dr Matt Nash |
| Bradford Royal Infirmary | Dr Sunita Seal |
| Broomfield, Chelmsford | Dr Ahmed Hassan |
| Calderdale Royal Hospital | Dr Karin Schwarz |
| Chelsea & Westminster | Dr Shu-Ling Chuang |
| Chesterfield & North Derbyshire Royal | Dr Aiwyne Foo |
| Colchester General | Dr Jo Anderson |
| Conquest | Dr Graham Whincup |
| Countess of Chester | Dr Stephen Brearey |
| Croydon University | Dr John Chang |
| Cumberland Infirmary | Dr Yee Aung |
| Darent Valley | Dr Abdul Hasib |
| Darlington Memorial Hospital | Dr Mehdi Garbash |
| Derriford Hospital | Dr Alex Allwood |
| Diana Princess of Wales | Dr Pauline Adiotomre |
| Doncaster Royal Infirmary | Dr Nigel Brooke |
| Dorset County | Dr Abby Deketelaere |
| East Surrey | Dr K Abdul Khader |
| Epsom General | Dr Ruth Shephard |
| Frimley Park | Dr Sanghavi Rekha |
| Furness General | Dr Anas Olabi |
| George Eliot | Dr Mukta Jain |
| Gloucester Royal | Dr Jennifer Holman |
| Good Hope | Dr Pinki Surana |
| Great Western | Dr Stanley Zengeya |
| Guy's & St Thomas' | Dr Geraint Lee |
| Harrogate District | Dr Sobia Balal |
| Hereford County | Dr Cath Seagrave |
| Hillingdon | Dr Tristan Bate |
| Hinchingbrooke | Dr Hilary Dixon |
| Homerton | Dr Narendra Aladangady |
| Hull Royal Infirmary | Dr Hassan Gaili |
| Ipswich | Dr Matthew James |
| James Cook University | Dr M Lal |

| | |
|---|---|
| James Paget | Dr Ambadkar |
| Kettering General | Dr Poornima Pandey |
| Kings College | Dr Ravindra Bhat |
| King's Mill | Dr Simon Rhodes |
| Kingston | Dr Vinay Pai |
| Lancashire Women and Newborn Centre | Dr Savi Sivashankar |
| Leeds | Dr Lawrence Miall |
| Leicester General | Dr Jonathan Cusack |
| Leicester Royal Infirmary | Dr Venkatesh Kairamkonda |
| Leighton | Dr Michael Grosdenier |
| Lincoln County | Dr Kollipara |
| Lister | Dr J  Kefas |
| Liverpool Women's | Dr Christopher Dewhurst |
| Luton & Dunstable | Dr Jennifer Birch |
| Macclesfield District General | Dr Gail Whitehead |
| Manor | Dr Krishnamurthy |
| Medway Maritime | Dr Ghada Ramadan |
| Milton Keynes General | Dr I Misra |
| Musgrove Park | Dr Chris Knight |
| New Cross | Dr Tilly Pillay |
| Newham General | Dr Imdad Ali |
| Nobles | Dr Prakash Thiagarajan |
| Norfolk & Norwich University | Dr Mark Dyke |
| North Devon District | Dr Michael Selter |
| North Manchester General | Dr P Kamath |
| North Middlesex University | Dr Neeraj Jain |
| Northumbria Specialist Emergency Care | Vivien Spencer |
| Northampton General | Dr Subodh Gupta |
| Northwick Park | Dr Richard Nicholl |
| Nottingham City | Dr Steven Wardle |
| Nottingham University | Dr Steven Wardle |
| Ormskirk District General | Dr Andreea Bontea |
| John Radcliffe | Dr Eleri Adams |
| Peterborough City | Dr Katharine McDevitt |
| Pilgrim | Dr Ajay Reddy |
| Pinderfields General (Pontefract General Infirmary) | Dr David Gibson |
| Poole General | Prof Minesh Khashu |
| Princess Alexandra | Dr Chinnappa Reddy |
| Princess Anne | Dr Mark Johnson |
| Princess Royal | Dr P Amess |
| Princess Royal (previously Royal Shrewsbury) | Dr Deshpande |
| Princess Royal University | Dr Elizabeth Sleight |
| Queen Alexandra | Dr Charlotte Groves |
| Queen Charlotte's | Dr Lidia Tyszcuzk |
| Queen Elizabeth, Gateshead | Dr Dennis Bosman |
| Queen Elizabeth, King's Lynn | Dr Glynis Rewitzky |
| Queen Elizabethl, Woolwich | Dr Olutoyin Banjoko |
| Queen Elizabeth the Queen Mother | Dr Bushra Abdul-Malik |

| | |
|---|---|
| Queen's Hospital, Burton on Trent | Dr Dominic Muogbo |
| Queen's Hospital, Romford | Dr Khalid Mannan |
| Queen's Hospital, Romford 2 | Dr Anand Shirsalkar |
| Rosie Maternity, Addenbrookes | Dr Angela D'Amore |
| Rotherham District General | Dr Shameel Mattara |
| Royal Albert Edward Infirmary | Dr Christos Zipitis |
| Royal Berkshire | Dr Peter De Halpert |
| Royal Bolton | Dr Paul Settle |
| Royal Cornwall | Dr Paul Munyard |
| Royal Derby | Dr John McIntyre |
| Royal Devon & Exeter | Dr David Bartle |
| Royal Hampshire County | Dr Lucinda Winckworth |
| Royal Lancaster Infirmary | Dr Joanne Fedee |
| Royal Oldham | Dr Natasha Maddock |
| Royal Preston | Dr Richa Gupta |
| Royal Stoke University | Dr Alison Moore |
| Royal Surrey County | Dr Ben Obi |
| Royal Sussex County | Dr Phil Amess |
| Royal United Hospital | Dr Stephen Jones |
| Royal Victoria Infirmary | Dr Naveen Athiraman |
| Russells Hall | Dr Mahadevan |
| Salisbury District | Dr Jim Baird |
| Scarborough General | Dr Kirsten Mack |
| Scunthorpe General | Dr Pauline Adiotomre |
| Southend | Dr Vineet Gupta |
| Southmead | Dr Alison Pike |
| St George's | Dr Charlotte Huddy |
| St Helier | Dr Salim Yasin |
| St Mary's, Isle of Wight | Dr Sian Butterworth |
| St Mary's, London | Dr Lidia Tyszczuzk |
| St Mary's, Manchester | Dr Ngozi Edi-Osagie |
| St Michael's | Dr Pamela Cairns |
| St Peter's | Dr Peter Reynolds |
| St Richard's | Dr Nick Brennan |
| Stepping Hill | Dr Carrie Heal |
| Stoke Mandeville | Dr Sanjay Salgia |
| Sunderland Royal | Dr Majd Abu-Harb |
| Tameside General | Dr Jacqeline Birch |
| The Jessop Wing, Sheffield | Dr Porus Bastani |
| The Royal Free | Dr Marice Theron |
| The Royal London | Dr Vadivelam Murthy |
| Torbay | Dr Siba Paul |
| Tunbridge Wells | Dr Hamudi Kisat |
| University College | Dr Giles Kendall |
| University Hospital Coventry | Dr Kate Blake |
| University Hospital Lewisham | Dr Ozioma Obi |
| University Hospital of North Durham | Dr Mehdi Garbash |
| University Hospital of North Tees | Dr Hari Kumar |
| Victoria Hospital, Blackpool | Dr Chris Rawlingson |

| | |
|---|---|
| Warrington | Dr Delyth Webb |
| Warwick | Dr Bird |
| Watford General | Dr Sankara Narayanan |
| West Cumberland | no lead |
| West Middlesex University | Dr Eleanor Hulse |
| West Suffolk | Dr Ian Evans |
| Wexham Park | Dr Rekha Sanghavi |
| Whipps Cross University | Dr Caroline Sullivan |
| Whiston | Dr Ros Garr |
| Whittington | Dr Wynne Leith |
| William Harvey | Dr Vimal Vasu |
| Worcestershire Royal | Dr Liza Harry |
| Worthing | Dr Katia Vamvakiti |
| Wythenshawe | Dr Ngozi Edi-Osagie |
| Yeovil District | Dr Megan Eaton |
| York District | Dr Sundeep Sandhu |

**Wales**

| **Hospital** | **Lead clinician** |
|---|---|
| Singleton | Dr Arun Ramachandran |
| Princess of Wales | Dr Kate Creese |
| Royal Gwent | Dr Sunil Reddy |
| Nevill Hall Hospital | Dr Sunil Reddy |
| Glan Clwyd l | Dr Ian Barnard |
| Wrexham Maelor | Dr Brendan Harrington |
| Ysbyty Gwynedd | Dr Mike Cronin |
| University Hospital of Wales | Dr Alok Sharma |
| Prince Charles | Dr Iyad Al-Muzaffar |
| Glangwili General | Dr Prem Pitchaikani |
| Withybush | Dr Vishwa Narayan |
