# Supplementary material for: Changes in neonatal admissions, care processes and outcomes in England and Wales during the COVID-19 pandemic: a whole population cohort study
Source: BMJ Open. 2021 Oct 1;11(10):e054410. doi: 10.1136/bmjopen-2021-054410 (PMC8488283; doi:10.1136/bmjopen-2021-054410)
Supplement: Supplementary data [file bmjopen-2021-054410supp002.pdf]

Supplementary Table. Changes during the Covid-19 period and the range of the corresponding changes in the previous years (* The direction or the difference is unique in the Covid-19 period.)

Group	Direction	Covid change	Pre-Covid range of changes
	Number of babies (N)		
Extremely preterm	Decrease*	–14	6, 68
Very preterm	—	–8	–24, 145
Moderate-to-late preterm	Decrease	–328	–212, 316
Full term	Decrease*	–1142	104, 1178
	Ethnic groups (N)		
	Asian		
Extremely preterm	—	4	–13, 27
Very preterm	—	–22	–36, 43
Moderate-to-late preterm	—	30	–6, 67
Full term	Decrease	–149	–79, 77
	Black		
Extremely preterm	—	3	–6, 21
Very preterm	—	–7	–43, 13
Moderate-to-late preterm	—	49	–53, 52
Full term	Increase	66	–64, 35
	White		
Extremely preterm	—	4	–22, 58
Very preterm	—	18	–17, 101
Moderate-to-late preterm	—	–123	–239, 182
Full term	Decrease	–218	–21, 365
	Other		
Extremely preterm	Decrease	–9	–5, 10
Very preterm	Decrease	–18	–15, 1
Moderate-to-late preterm	—	–6	–13, 37
Full term	—	–3	–25, 63

Group	Direction	Covid change	Pre-Covid range of changes
		Ethnic groups (%)	
		Asian	
Extremely preterm	—	0.85	–3.75, 4.48
Very preterm	—	–2.07	–2.76, 2.96
Moderate-to-late preterm	—	0.87	–0.40, 1.17
Full term	—	–1.28	–1.28, 0.37
		Black	
Extremely preterm	—	0.65	–1.57, 3.63
Very preterm	—	–0.55	–3.88, 1.51
Moderate-to-late preterm	Increase	1.23	–0.90, 0.83
Full term	Increase	1.03	–0.63, 0.24
		White	
Extremely preterm	—	0.64	–4.92, 3.23
Very preterm	Increase	4.53	–2.37, 4.04
Moderate-to-late preterm	—	–2.01	–2.31, 0.54
Full term	—	0.05	–0.66, 1.23
		Other	
Extremely preterm	Decrease	–2.13	–1.25, 1.15
Very preterm	Decrease	–1.91	–1.14, 0.06
Moderate-to-late preterm	—	–0.09	–0.23, 0.55
Full term	—	0.18	–0.24, 0.59
		CS. emergency (%)	
Extremely preterm	—	0.28	–2.53, 5.80
Very preterm	—	0.53	–5.21, 2.82
Moderate-to-late preterm	Increase	2.05	–1.51, 1.71
Full term	—	–0.12	–0.28, 0.84
		CS. elective (%)	
Extremely preterm	—	0.46	–2.06, 1.30
Very preterm	Decrease	–2.30	–1.27, 1.95
Moderate-to-late preterm	Increase	0.51	–1.06, 0.35
Full term	—	0.99	–0.77, 1.10

Group	Direction	Covid change	Pre-Covid range of changes
	Mortality (%)		
	Died at age 1–7 days		
Extremely preterm	—	–0.72	–0.98, 1.99
Very preterm	—	0.59	–0.36, 0.66
Moderate-to-late preterm	—	0.04	–0.03, 0.24
Full term	—	0.05	0.01, 0.11
	Died at age 8–28 days		
Extremely preterm	—	–0.36	–1.35, 0.40
Very preterm	Decrease	–0.96	–0.12, 0.31
Moderate-to-late preterm	—	0.06	–0.06, 0.10
Full term	—	0.04	–0.03, 0.08
	Transfer (%)		
	Downward		
Extremely preterm	—	0.37	–1.07, 0.60
Very preterm	—	–1.12	–1.22, 1.14
Moderate-to-late preterm	Decrease	–0.30	–0.25, 0.20
Full term	Decrease	–0.09	–0.06, 0.02
	Horizontal		
Extremely preterm	—	0.20	–0.55, 1.00
Very preterm	—	–0.69	–0.79, 0.91
Moderate-to-late preterm	—	–0.12	–0.27, 0.21
Full term	—	0.03	–0.10, 0.19
	Upward		
Extremely preterm	Increase	3.58	–6.14, 2.34
Very preterm	—	0.59	–0.42, 1.16
Moderate-to-late preterm	Increase	0.73	–0.18, 0.23
Full term	Increase	0.67	–0.07, 0.23

Group	Direction	Covid change	Pre-Covid range of changes
		Number of babies (N)	
All preterm	Decrease	–350	–26, 479
Full term	Decrease*	–1142	104, 1178
		Ethnic groups (%)	
		Asian	
All preterm	Increase	3.35	–1.67, 3.25
Full term	Decrease	–3.35	–3.25, 1.67
		Black	
All preterm	—	–0.05	–4.54, 2.96
Full term	—	0.05	–2.96, 4.54
		White	
All preterm	—	0.28	–1.41, 0.48
Full term	—	–0.28	–0.48, 1.41
		Other	
All preterm	—	–2.73	–3.00, 1.77
Full term	—	2.73	–1.77, 3.00
		CS. emergency (%)	
All preterm	—	1.70	–1.48, 1.82
Full term	—	–0.12	–0.28, 0.84
		CS. elective (%)	
All preterm	—	0.02	–0.93, 0.49
Full term	—	0.99	–0.77, 1.10
		Mortality (%)	
		Died at age 1–7 days	
All preterm	—	0.10	–0.09, 0.17
Full term	—	0.05	0.01, 0.11
		Died at age 8–28 days	
All preterm	Decrease	–0.10	–0.09, 0.05
Full term	—	0.04	–0.03, 0.08

Group	Direction	Covid change	Pre-Covid range of changes
		Severe brain injury (N)	
Extremely preterm	—	5	–8, 24
Very preterm	Decrease	–1	0, 24
Moderate-to-late preterm	—	8	–23, 20
Full term	—	21	–6, 51
		Therapeutic hypothermia (N)	
Extremely preterm	—	2	–2, 4
Very preterm	—	–2	–4, 0
Moderate-to-late preterm	—	1	–8, 13
Full term	—	9	–6, 45
		Bronchopulmonary dysplasia (N)	
Extremely preterm	—	3	–12, 38
Very preterm	—	–14	–14, 50
Moderate-to-late preterm	—	12	–32, 25
Full term	Not applicable		
		Necrotising enterocolitis (N)	
Extremely preterm	—	–6	–9, 5
Very preterm	Decrease	–13	–10, 2
Moderate-to-late preterm	—	1	–7, 8
Full term	—	0	–3, 0
		Antenatal steroids (N)	
Extremely preterm	Decrease*	–14	11, 65
Very preterm	—	–25	–47, 139
Moderate-to-late preterm	—	–178	–230, 269
Full term	Decrease	–46	–20, 72
		Intubation at resuscitation (N)	
Extremely preterm	Decrease	–25	–2, 51
Very preterm	—	15	–30, 57
Moderate-to-late preterm	—	–13	–32, 19
Full term	—	20	–41, 46

Group	Direction	Covid change	Pre-Covid range of changes
		Surfactant (*N*)	
Extremely preterm	—	9	3, 54
Very preterm	—	–1	–39, 62
Moderate-to-late preterm	—	–32	–43, 60
Full term	—	14	–30, 43
	Surgery for patent ductus arteriosus (*N*)		
Extremely preterm	—	–1	–9, 4
Very preterm	Increase	2	–5, 1
Moderate-to-late preterm	—	0	–1, 2
Full term	—	0	–2, 1
		Born at a level 3 unit (*N*)	
Extremely preterm	Decrease*	–40	3, 71
Very preterm	—	22	–82, 85
Moderate-to-late preterm	—	–44	–74, 215
Full term	—	–327	–363, 822
	Mother's milk exclusive at discharge (*N*)		
Extremely preterm	Decrease	–27	–19, 25
Very preterm	—	41	–21, 73
Moderate-to-late preterm	Decrease*	–73	51, 169
Full term	Decrease*	–622	251, 629

Group	Direction	Covid change	Pre-Covid range of changes
		Severe brain injury (%)	
Extremely preterm	—	1.49	–1.78, 1.49
Very preterm	—	–0.05	–0.49, 1.54
Moderate-to-late preterm	—	0.19	–0.41, 0.34
Full term	Increase	0.33	–0.22, 0.31
		Therapeutic hypothermia (%)	
Extremely preterm	—	0.36	–0.41, 0.60
Very preterm	—	–0.17	–0.32, 0.00
Moderate-to-late preterm	—	0.04	–0.14, 0.21
Full term	—	0.20	–0.14, 0.31
		Bronchopulmonary dysplasia (%)	
Extremely preterm	—	2.46	–1.76, 3.14
Very preterm	Decrease	–1.02	–0.97, 3.01
Moderate-to-late preterm	Increase	0.59	–0.72, 0.07
Full term		Not applicable	
		Necrotising enterocolitis (%)	
Extremely preterm	—	–0.88	–2.17, 0.41
Very preterm	Decrease	–1.12	–0.86, 0.10
Moderate-to-late preterm	—	0.02	–0.12, 0.11
Full term	—	0.00	–0.03, 0.00
		Antenatal steroids (%)	
Extremely preterm	—	–0.02	–1.03, 3.74
Very preterm	—	–1.52	–1.92, 1.64
Moderate-to-late preterm	—	–0.08	–1.00, 1.36
Full term	—	–0.02	–0.28, 0.39
		Intubation at resuscitation (%)	
Extremely preterm	—	–2.60	–2.73, 1.21
Very preterm	—	1.46	–3.31, 1.78
Moderate-to-late preterm	—	–0.08	–0.48, 0.37
Full term	Increase	0.29	–0.52, 0.15
		Surfactant (%)	
Extremely preterm	Increase	2.46	–1.57, 1.17
Very preterm	—	–0.49	–2.60, 2.13
Moderate-to-late preterm	—	–0.58	–0.93, 0.95
Full term	—	0.35	–0.25, 0.47

Group	Direction	Covid change	Pre-Covid range of changes
	Surgery for patent ductus arteriosus (%)		
Extremely preterm	—	–0.13	–1.78, 0.67
Very preterm	Increase	0.17	–0.39, 0.07
Moderate-to-late preterm	—	0.00	–0.02, 0.03
Full term	—	0.00	–0.02, 0.01
	Born at a level 3 unit (%)		
Extremely preterm	Decrease	–5.20	–1.36, 5.25
Very preterm	—	2.24	–5.19, 3.00
Moderate-to-late preterm	Increase	1.44	–0.99, 1.22
Full term	—	0.96	–2.60, 1.80
	Mother's milk exclusive at discharge (%)		
Extremely preterm	—	–1.85	–5.00, 1.87
Very preterm	Increase	4.23	–2.92, 3.33
Moderate-to-late preterm	—	0.29	0.00, 2.36
Full term	Decrease	–2.32	–0.14, 3.96
