# Supplementary material for: Changes in neonatal admissions, care processes and outcomes in England and Wales during the COVID-19 pandemic: a whole population cohort study
Source: BMJ Open. 2021 Oct 1;11(10):e054410. doi: 10.1136/bmjopen-2021-054410 (PMC8488283; doi:10.1136/bmjopen-2021-054410)
Supplement: Author's manuscript [file bmjopen-2021-054410.draft_revisions.pdf]

BMJ Open

BMJ Open is committed to open peer review. As part of this commitment we make the peer review history of every article we publish publicly available.

When an article is published we post the peer reviewers' comments and the authors' responses online. We also post the versions of the paper that were used during peer review. These are the versions that the peer review comments apply to.

The versions of the paper that follow are the versions that were submitted during the peer review process. They are not the versions of record or the final published versions. They should not be cited or distributed as the published version of this manuscript.

BMJ Open is an open access journal and the full, final, typeset and author-corrected version of record of the manuscript is available on our site with no access controls, subscription charges or pay-per-view fees (http://bmjopen.bmj.com).

If you have any questions on BMJ Open's open peer review process please email info.bmjopen@bmj.com

BMJ Open

Changes in neonatal admissions, care processes and outcomes in England and Wales during the COVID-19 pandemic

Journal:	*BMJ Open*
Manuscript ID	bmjopen-2021-054410
Article Type:	Original research
Date Submitted by the Author:	14-Jun-2021

Complete List of Authors:	Greenbury, Sam; Imperial College London, Section of Neonatal Medicine, School of Public Health, Faculty of Medicine; Imperial College London, Institute for Translational Medicine and Therapeutics Data Science Group<br>Longford, Nicholas; Imperial College London, Section of Neonatal Medicine<br>Ougham, Kayleigh; Imperial College London, Section of Neonatal Medicine, School of Public Health, Faculty of Medicine<br>Angelini, Elsa; Imperial College London, Institute for Translational Medicine and Therapeutics Data Science Group<br>Battersby, Cheryl; Imperial College London, NHS England<br>Uthaya, Sabita; Imperial College London, Section of Neonatal Medicine, School of Public Health, Faculty of Medicine<br>Modi, Neena; Imperial College London, Medicine

Keywords:	Neonatal intensive & critical care < INTENSIVE & CRITICAL CARE, NEONATOLOGY, PUBLIC HEALTH

SCHOLARONE™
Manuscripts

I, the Submitting Author has the right to grant and does grant on behalf of all authors of the Work (as defined in the below author licence), an exclusive licence and/or a non-exclusive licence for contributions from authors who are: i) UK Crown employees; ii) where BMJ has agreed a CC-BY licence shall apply, and/or iii) in accordance with the terms applicable for US Federal Government officers or employees acting as part of their official duties; on a worldwide, perpetual, irrevocable, royalty-free basis to BMJ Publishing Group Ltd ("BMJ") its licensees and where the relevant Journal is co-owned by BMJ to the co-owners of the Journal, to publish the Work in this journal and any other BMJ products and to exploit all rights, as set out in our licence.

The Submitting Author accepts and understands that any supply made under these terms is made by BMJ to the Submitting Author unless you are acting as an employee on behalf of your employer or a postgraduate student of an affiliated institution which is paying any applicable article publishing charge ("APC") for Open Access articles. Where the Submitting Author wishes to make the Work available on an Open Access basis (and intends to pay the relevant APC), the terms of reuse of such Open Access shall be governed by a Creative Commons licence – details of these licences and which Creative Commons licence will apply to this Work are set out in our licence referred to above.

Other than as permitted in any relevant BMJ Author's Self Archiving Policies, I confirm this Work has not been accepted for publication elsewhere, is not being considered for publication elsewhere and does not duplicate material already published. I confirm all authors consent to publication of this Work and authorise the granting of this licence.

For peer review only

Changes in neonatal admissions, care processes and outcomes in England and Wales during the COVID-19 pandemic

Sam F Greenbury PhD [1, 2], Nicholas T Longford PhD [1], Kayleigh Ougham MSc [1], Elsa D Angelini PhD [2], Cheryl Battersby FRCPH [1], Sabita Uthaya FRCP [1], Neena Modi FMedSci [1]

1 Section of Neonatal Medicine, School of Public Health, Faculty of Medicine, Chelsea and Westminster Hospital campus, Imperial College London, London, UK

2 National Institute for Health Research Imperial Biomedical Research Centre, Institute for Translational Medicine and Therapeutics Data Science Group, Imperial College London, London, UK

Correspondence to Professor N Modi, Section of Neonatal Medicine, School of Public Health, Faculty of Medicine, Imperial College London, Chelsea and Westminster Hospital campus, 369 Fulham Road, London SW10 9NH, UK; email: n.modi@imperial.ac.uk; tel: +44 (0) 203 315 5101

Manuscript word count: 3515

Keywords: neonatal; admissions; COVID-19; births; preterm; full-term; pandemic; healthcare; outcomes; National Neonatal Research Database

For peer review only

For peer review only - http://bmjopen.bmj.com/site/about/guidelines.xhtml

Abstract

300w

Objectives: The COVID-19 pandemic instigated multiple societal and healthcare interventions with potential to affect perinatal practice. We evaluated population-level changes in preterm and full-term admissions to neonatal units, care processes, and outcomes.

Setting and participants: Admissions to National Health Service neonatal units in England and Wales from 2012-2020.

Main outcomes measures: Admissions by gestational age, ethnicity, and Index of Multiple Deprivation, and key care processes and outcomes.

Design: Information from the UK National Neonatal Research Database that holds quality-assured data extracted from routine Electronic Patient Records. We calculated differences in numbers and rates between April-June 2020 (spring) the first three months of national lockdown (COVID period), and December 2019-February 2020 (winter), prior to introduction of mitigation measures, and compared them with the corresponding differences in the seven previous years. We considered the COVID period highly unusual if the spring-winter difference was smaller or larger than all previous corresponding differences, and calculated the level of confidence in this conclusion.

Results: Marked fluctuations occurred in all measures over the eight years with several highly unusual changes during the COVID period. Total admissions fell, having risen over all previous years (COVID difference: -1492; previous seven-year difference range: +100, +1617; p<0.001); full-term Black admissions rose (+66; -64, +35; p<0.001) whereas Asian (-137; -14, +101; p<0.001) and White (-319; -235, +643: p<0.001) admissions fell. Transfers to higher and lower designation neonatal units increased (+129; -4, +88; p<0.001) and decreased (-47; -25, +12; p<0.001), respectively. Total preterm admissions decreased (-350; -26, +479; p<0.001). The fall in extremely preterm admissions was most marked in the two lowest socio-economic quintiles.

Conclusions: Our findings indicate substantial changes occurred in care pathways and clinical thresholds, with disproportionate effects on Black ethnic groups, during the immediate COVID-19 period, and raise the intriguing possibility that non-healthcare interventions may reduce extremely preterm births.

For peer review only - http://bmjopen.bmj.com/site/about/guidelines.xhtml

What is already known on this topic

- There have been 8 previous studies involving small numbers of extremely and very preterm infants, 7 of which suggested preterm births and admissions decreased in association with the onset of the COVID-19 pandemic, and one that found no change

What this study adds

- Our large whole population study identified a highly unusual fall in both preterm and full-term admissions to neonatal units in England and Wales during the immediate COVID-19 national lockdown, and a highly unusual rise in inter-hospital transfers of mature babies to higher level units
- Total admissions fell by 1492 between April-June 2020 and December 2019-February 2020 but this masked a highly unusual rise in Black full-term admissions, in contrast with other ethnic groups
- The fall in extremely preterm admissions was most marked in the most deprived socio-economic groups, was sustained into the months July-September 2020, and was not explained by a rise in stillbirths

.

Strengths and limitations of this study

- Our study is a complete population evaluation that included all admissions to NHS neonatal units in England and Wales over an eight-year period
- We assessed full-term, as well as extremely preterm, very preterm, and moderate-to-late preterm groups individually
- All previous studies have compared a COVID period with earlier periods with the implicit assumption that COVID-19 is the only agent likely to have influenced outcomes; however we show clearly there have been marked fluctuations in outcomes over time, hence assessed differences between the first national COVID-19 lockdown period and the preceding quarter, and compared these with corresponding differences in the previous seven years
- A limitation of our approach is that our measure of exceptionality may be too conservative, potentially hindering detection of a COVID-19 effect
- We were unable to evaluate national data on births by gestational age directly as these were not available

For peer review only - http://bmjopen.bmj.com/site/about/guidelines.xhtml

Introduction

The COVID-19 pandemic, the consequence of the emergence of a novel virus, SARS-CoV-2, has had potential to affect maternal and newborn health in multiple ways. In the United Kingdom (UK), the first full national lockdown commenced on March 23rd 2020 (1). This included requiring people to stay at home except for essential reasons, closure of public venues and all non-essential businesses, and prohibition of public gatherings. The national lockdown, and other policies implemented in an attempt to mitigate the spread of the virus, led to changes in hospital and general practitioner care, and alterations in environmental and societal factors. Thus, air quality improved in many highly populated urban areas (2), but reports of mental stress, domestic violence and child abuse, increased (3, 4). On February 18th 2020, NHS England advised the UK public not to contact their general practitioners, or go to hospital Accident and Emergency Departments, but instead to contact the NHS111 online and telephone service for medical advice (5). Within hospitals, In addition to the direct consequences of infection, the abrupt onset of the pandemic necessitated rapid implementation of changes in healthcare processes based on standard infection-control policies, without specific knowledge of the transmissibility, pathogenicity and epidemiology of the novel virus. The rapidity of spread led to re-deployment of healthcare staff and prioritised allocation of resources, such as personal protective equipment, to areas of greatest need.

There have been eight reports evaluating preterm births in relation to the onset of the pandemic; seven describe a reduction (5-12), and one no change (13). The spontaneous onset of preterm labour is associated with a number of factors, including infection, systemic illness, severe stress, and physical injury. From an epidemiological perspective, seasonal effects, socio-economic factors and population characteristics also affect the preterm birth rate (14). The pandemic might have additionally have influenced rates of elective Caesarean section, with and without medical indication, which are an iatrogenic cause of late preterm births, and a well-recognised cause of respiratory and other problems that lead to neonatal unit admission (15). However, the incidence of births by elective Caesarean section varies by population demographics, across healthcare systems and with time. Thus, for many reasons, identifying any causal determinants of preterm birth is problematic.

Our aim in this study was to determine if any "highly unusual" changes in admissions to neonatal units in England and Wales, care processes and outcomes occurred following the start of the first national lockdown. Recognising the marked fluctuations in these measures over time, we determined if changes in the immediate COVID-19 period, namely April to June 2020, when compared with the preceding quarter, December 2019 to February 2020, were highly unusual in relation to differences between equivalent periods over the preceding seven years. We also determined if any highly unusual changes persisted into the period July to September 2020.

Methods

The study was undertaken under approval from the Health Research Authority and Health and Care Research Wales, and with the agreement of all NHS neonatal units in England and Wales. Contributing neonatal units and their clinical leads are listed in Supplementary table S1.

For peer review only - http://bmjopen.bmj.com/site/about/guidelines.xhtml

Data sources

Neonatal admissions: We examined the entire population of babies admitted to National Health Service (NHS) neonatal units in England and Wales over the period December 2012 to September 2020. We obtained information on admissions, including the numbers of suspected and proven SARS-CoV-2 cases for mothers and babies, over the study period, from the National Neonatal Research Database (NNRD). This is a national information asset containing detailed clinical information extracted from the electronic patient records of all admissions to NHS neonatal units (16). Data are quality-assured to a research standard (17). As the care of preterm and sick neonates outside of NHS neonatal units is exceptionally rare in the UK, the data comprise the complete population of eligible infants. Neonatal care in England and Wales is delivered in a networked operational model, with babies transferred to higher or lower designation neonatal units according to care needs. Data management procedures for the NNRD therefore include linking episodes of care across neonatal units to provide a complete, single, record from admission to discharge for each baby. No additional data management procedures were undertaken for this study.

Total live and stillbirths: We obtained data on stillbirths and total livebirths from the UK Office for National Statistics (18).

Outcomes

We categorised admissions by gestational age as defined by the World Health Organisation (extremely preterm GA1: <28^{+0}; very preterm GA2: 28^{+0} to 31^{+6}; moderate to late preterm GA3: 32^{+0} to 36^{+6}; and full term GA4: ≥37^{+0} weeks^{+days}), ethnicity, using collapsed NHS codes (Asian; Black; White; Mixed/Other) (19), and Index of Multiple Deprivation (IMD) quintile through mapping of the maternal Lower-layer Super Output Area (LSOA) (20). The IMD is the official measure of relative deprivation for small areas in England, formed by combining information from seven weighted domains (income; employment; education, skills and training; health and disability; crime; housing and services; living environment) to produce an overall measure of deprivation. The LSOA defines an area of similar population size, with an average of approximately 1,500 residents or 650 households.

In addition to admissions, we evaluated a range of care processes and key neonatal outcomes. These were: postnatal transfers (downward, from a higher to lower designation neonatal unit; horizontal, to an equivalent designation neonatal unit; upward, from a lower to higher designation neonatal unit); mode of delivery (elective Caesarean section; emergency Caesarean section); all-cause mortality (early neonatal (days 1-7); late neonatal (days 8-28)); intubation at resuscitation, surfactant administration, ligation of patent ductus arteriosus, bronchopulmonary dysplasia (defined as any respiratory support or supplemental oxygen at 36 weeks postmenstrual age), death from or surgery for necrotising enterocolitis, severe brain injury (defined as any seizures, hypoxic ischaemic encephalopathy, intracranial haemorrhage, white matter injury, stroke, central nervous system infection or kernicterus), therapeutic hypothermia, and breast-feeding at discharge.

Analyses

We compared admissions, processes and outcomes for the initial COVID-19 period April-June 2020 (spring) with the preceding period December 2019-February 2020 (winter), and contrasted these differences with the differences for the corresponding pairs of periods in the preceding years from 2013 (i.e. seven sets of paired differences). We made an a priori

For peer review only - http://bmjopen.bmj.com/site/about/guidelines.xhtml

decision to exclude March 2020, as this represented a period of variable response to the pandemic. We also considered whether any changes between winter and spring 2020 were sustained into July-September (summer). We did not utilise data prior to 2013 as complete data for England and Wales were not available. We excluded ethnicity from the analysis for Wales, as these data were not available for 2020. We evaluated differences in absolute numbers as well as differences in rates.

We defined the change in each measure during the initial COVID period, April-June 2020 (spring), as "highly unusual" if the difference with the period December 2019-February 2020 (winter) was smaller or larger than all previous corresponding differences. We adopted an empirical Bayes approach to provide a post hoc measure of confidence, or relative strength in the estimate of the difference in rates (21). For each measure and gestational age category, we held-out the two three-month periods for the COVID difference and used the 14 pre-COVID differences to estimate the background against which to consider the former. For the fourteen pre-COVID differences we identified posterior distributions over the binomial probabilities, approximating them with Gaussian distributions by moment matching and applying shrinkage assuming the individual three-month rates are drawn from a common distribution. We then drew 10,000 independent samples from the fourteen posterior distributions to yield a posterior distribution for each of the seven spring-winter differences. For the seven sets of 10,000 posterior samples we evaluated the proportion that did not meet our criterion for "highly unusual". This provides an estimate of the probability (the p-value) that the COVID period was not "highly unusual". We used a 0.05 threshold as a measure of the strength of the evidence for this conclusion.

We present results in tables and figures showing the periods December-February, April-June and July-September by year, highlighting any highly unusual changes.

Results

There were 729,363 admissions to neonatal units in England and Wales over the period December 2012 to September 2020. We identified marked fluctuations in all measures over the eight years. However, during the COVID period April-June 2020, in comparison with the preceding period December-February, there were several changes that were both highly unusual and met our strength of evidence threshold (Table 1). Admissions fell (COVID period difference: total -1492; previous seven-year difference range: +100, +1617; p<0.001; full-term: -1142; +104, +1178; p<0.001; preterm: -350; -26, +477; p<0.001). The absolute number of admissions in all preterm gestational age categories over April-June 2020 (7882) was also the lowest for any April-June or December-February period over the previous seven years (range 8505, 9184). The fall in GA1 (extremely preterm) and GA2 (very preterm) admissions, the most immature babies, continued into the period July to September 2020, unlike GA3 (moderate-to-late preterm) and GA4 (full-term) which rose again (Fig 1).

There were highly unusual spring-winter falls in GA1 admissions in IMD quintile 1, and GA2 admissions in IMD quintiles 1 and 2, though only the latter had a p-value below 0.05 (-41; -20, +59; p=0.036). There were highly unusual falls in GA4 admissions in IMD quintiles 3, 4 and 5, and additionally in GA3 admissions in IMD 5 (Fig 2). The fall in GA1 admissions continued into the period July-September. Full-term Black ethnicity admissions rose (+66; -64, +35; p<0.001) in spring, and then fell in the summer (Fig 3), in contrast to spring reductions in total Asian (-137; -14, +101; p<0.001) and total White (-319; -235, +643: p<0.001) groups

For peer review only - http://bmjopen.bmj.com/site/about/guidelines.xhtml

(Table 1). Transfers to higher designation neonatal units increased (+129; -4, +88; p<0.001). Transfers to lower designation neonatal units decreased (-47; -25, +12; p<0.001).

There were other highly unusual changes. There was a decrease in the number of GA2 babies born by elective Caesarean section (-27; -17, +34; p=0.035). The number of GA1 babies born in a hospital with a level 3 (neonatal intensive care) unit fell (-40; +3, +71; p=0.027). The percentage of GA2 babies having surgery for necrotising enterocolitis fell (-1.1%; -0.9%, +0.1%; p=0.017). Breast-feeding at discharge fell in GA3 babies (-202; -91, +170; p=0.031; -1.7%; -1.1%, +1.5%; p=0.047), but rose in GA4 babies (+1.4%; -1.2%, +1.0%; p=0.031).

There were also changes that fulfilled our criteria for "highly unusual" but did not meet our strength of evidence threshold, and where numbers were small or where a similar sized effect had occurred during the preceding seven years, casting uncertainty on their relevance. The number of GA4 babies born by emergency Caesarean section fell (-186; +45, +500); the percentage requiring intubation at resuscitation rose (+ 0.3%; -0.5, +0.15) as did the proportion with severe brain injury (+0.3%; -0.2, +0.3). The percentage of GA1 babies receiving surfactant (+2.5%; -1.6, +1.2) and the number and percentage of GA2 babies receiving surgery for patent ductus arteriosus (N: +2; -5, +1; %: +0.2%; -0.4, +0.1) rose. The percentage of GA3 babies developing bronchopulmonary dysplasia fell (+0.6%; -0.7, +0.1). We identified no highly unusual changes in antenatal steroid use, horizontal transfers, therapeutic hypothermia or early and late neonatal mortality.

We show the number of suspected and confirmed cases of COVID-19 in mothers and babies over the periods December 2019-February 2020, April 2020-June 2020 and July-September 2020 in Table 2. Using Office for National Statistics data, we show changes in stillbirths and livebirths for England and Wales over the study period; these do not suggest a highly unusual change occurred over April-June 2020 (Fig 4).

Discussion

We identified highly unusual changes in key perinatal measures during the immediate period of the first national UK lockdown, although the number of confirmed cases of COVID-19 in babies admitted to neonatal units, and their mothers, was small. Our study included all admissions to NHS neonatal units in England and Wales over an eight-year period. We assessed all preterm and full-term admissions as well as extremely preterm, very preterm, and moderate-to-late preterm groups individually, as degree of immaturity has a cardinal influence upon care pathways and morbidities. In view of known seasonal fluctuations in births, we assessed the difference between the immediate period of national COVID-19 lockdown with the preceding quarter, excluding a priori the entire month of March 2020, and compared them with differences in the corresponding epochs of the previous seven years.

We found a highly unusual fall in full-term admissions during the immediate COVID-19 period. This was not due to a fall in total births, or a reduction in elective Caesarean sections, following which infants are more likely to require neonatal unit admission than those born vaginally (15). This suggests a rise in the clinical threshold for the admission of mature babies to neonatal units occurred during the immediate COVID-19 lockdown. Despite the fall in admissions, there was a highly unusual increase in transfers of moderate-to-late preterm and full-term babies to a higher designation neonatal unit. Upward transfer of mature babies is usually only undertaken if higher intensity care is required, suggesting the number with serious illness increased substantially. In this context, the increase in the proportion of full-term babies born by emergency Caesarean section, requiring intubation for resuscitation, and

For peer review only - http://bmjopen.bmj.com/site/about/guidelines.xhtml

with severe brain injury, should be noted. These changes fulfilled our criteria for highly unusual, although numbers were small and our strength of evidence threshold was not reached. A further notable finding was that the fall in full-term admissions masked a highly unusual increase in the number of admissions of full-term babies of Black ethnicity, contrasting with a decrease in Asian and White ethnic groups. Taken together, our data indicate greater likelihood of late presentation and delayed delivery of mature babies in fetal distress, in accord with the known marked reduction in all healthcare-seeking behaviours with the onset of the pandemic (22, 23), and greater adverse impact upon Black communities (24, 25).

We found evidence of other perturbations to neonatal care pathways. It is a UK standard of care to deliver extremely preterm infants in a hospital with a level 3 (neonatal intensive care) neonatal unit (26). However, during the immediate COVID period there was a highly unusual decrease in the number of extremely preterm babies born in hospitals with a level 3 neonatal unit. This indicates that obstetric *in utero* transfers (transfers of mothers at risk of extremely preterm delivery to a tertiary centre) were less likely. The fall in total admissions meant it was important to evaluate the proportion of babies experiencing a particular outcome. We identified changes that though fulfilling our criteria for highly unusual, and meeting our strength of evidence threshold, were small, and may have occurred by chance. These included a decrease in the proportion of very preterm babies receiving surgery for necrotising enterocolitis and an increase in the proportion of full-term babies breast-feeding at discharge.

We also identified a highly unusual fall in all preterm admissions. The numbers of moderate-to-late preterm babies dominate the preterm category, and a fall in their admission numbers may, as with full-term babies, reflect a rise in clinical thresholds. However, we also found a highly unusual fall in extremely preterm admissions, those born below 28 weeks gestation, a change that appeared confined to the two lowest IMD quintiles representing the most deprived groups. In both, the fall continued into the period July-September 2020. The absolute numbers of extremely preterm babies, even in a whole population dataset, are small, hence it is unsurprising that even though highly unusual, the fall did not meet our stringent statistical threshold. There have however been seven previous reports of a fall in preterm births during the immediate COVID-19 period, though all involved substantially smaller numbers than our study (6-12). Berghella et al compared records from a single hospital in northeast United States over March 1 to July 31 2020, with the same period in 2019 (6). They identified seven births below 28 weeks gestation in 2020, compared with fourteen in the previous year. Philip et al compared births at a regional hospital in Ireland over Jan 1-April 30, 2020 with the same period of the preceding nineteen years, identifying only three very and extremely low birthweight infants compared to a predicted number of eight (7). However, Ireland implemented lockdown measures in early March, not in early January, weakening the inference of a temporal association. Been et al used a difference-in-regression-discontinuity approach to study the impact on preterm births of COVID-19 mitigation measures introduced at three points in March 2020 in the Netherlands. They identified a statistically significant reduction only in moderate-to-late preterm births and only in relation to the first time-point (8). Hedermann et al compared the period March 12 to April 14, 2020 with the average rate in Denmark over the previous five years (9). They identified only fifty-eight extremely preterm births over the five-year period and noted extremely preterm births were significantly lower in 2020, but not very or moderate-to-late preterm births. They were unable to exclude the possibility of a corresponding rise in late abortions or

For peer review only - http://bmjopen.bmj.com/site/about/guidelines.xhtml

stillbirths. Matheson et al studied births in three maternity hospitals in Melbourne, Australia, identifying nine extremely preterm births over July-September 2020, compared with twenty during the same period in 2019 (10). Lemon et al describe a decrease in preterm births in a single US hospital limited to White women from more advantaged neighbourhoods (11). Maeda et al studied records from 186 Japanese acute care hospitals noting a decrease in preterm births but the 95% confidence interval for the adjusted incidence rate ratios included or were close to one (below 34 weeks gestation: 0.71; 95% CI, 0.50 to 1.00; below 37 weeks: 0.85; 95% CI, 0.74 to 0.98) and the extent of population coverage is not known (12). Handley et al noted no decrease in preterm births in two Philadelphia hospitals (13).

All these studies compared a COVID period with earlier periods. In such a direct comparison there is an implicit assumption that COVID-19 is the only agent likely to have influenced the outcome. However, as we show, there have been marked fluctuations in outcomes over previous years. As the onset and duration of other influences is unknown, subsuming them into the residual error term of a model risks deriving a flawed estimate. In contrast to these studies, we considered the *differences* between three-month pre- and post-COVID periods and compared them to the corresponding three-month differences over seven previous years. By comparing differences we are able to assess the strength of a change during the COVID period taking other, unknown, influences into account. We acknowledge, however, that a limitation of our approach is that our measure of exceptionality may be too conservative, potentially hindering detection of a COVID-19 effect.

We identified a fall in extremely preterm admissions over April-June 2020 in comparison to December 2019-February 2020, whereas in all previous seven years the number rose over corresponding periods. In the UK, all extremely preterm babies are admitted to an NHS neonatal unit, hence the fall likely reflects a genuine reduction in live births in this gestational age group. Though a small study from a single London hospital, employing a before and after approach, suggested stillbirths rose during the immediate COVID period (27), this is not supported by data from the Office for National Statistics. Our finding that the highly usual reduction in extremely preterm admissions during the immediate COVID national lockdown occurred in the most deprived socio-economic groups and was sustained into the following three months, is intriguing. Globally, preterm birth rates are increasing, with a strong association with poverty, disadvantage and deprivation (28). Attempts to lower the preterm birth rate have remained stubbornly resistant to a range of medical interventions over the years, from widespread use of tocolytics, bedrest, cervical cerclage, vaginal progesterone, and enhanced surveillance. Thus the possibility that non-healthcare related interventions may be effective is important.

In conclusion, our observation of a fall in extremely preterm admissions during the immediate period of national COVID-19 lockdown, sustained in lower socio-economic groups into the subsequent three months, requires corroboration, and we hope data will be forthcoming from other large, population-based birth cohorts. Our findings should also provide impetus to study the effects on preterm births of public health interventions, such as improved air quality, reduced exposure to crowded environments, and altered working during the second trimester of pregnancy, and their interactions with other trigger events. The reasons for the fall in admissions of more mature babies are more likely to be related to changes in clinical thresholds. Together with evidence of perturbations in care pathways, these findings justify consideration of preparedness and public messaging during national crises adding weight to calls for an official COVID-19 inquiry into UK Government actions (29), such as the

For peer review only - http://bmjopen.bmj.com/site/about/guidelines.xhtml

recommendation to rely upon the call service NHS111 for medical advice (30), that has now been agreed but deferred until the spring of 2022 (31). Finally, the highly unusual rise in admissions of full-term Black ethnicity babies, contrasted with a fall in all other ethnic groups, adds to the growing evidence of a disproportionately higher adverse impact upon this demographic group and speaks to the moral imperative to address ethnic and socio-economic health disparities urgently, as well as growing calls for investment in research to improve maternal and newborn health (32).

Acknowledgements

We gratefully acknowledge the contribution of all UK Neonatal Collaborative neonatal units to the National Neonatal Research Database, Mr Richard Colquhoun, Research Manager and data analysts Victor Banda and Julia Lanoue.

Contributor statement

All authors had full access to all study data and take responsibility for the integrity of the data, the accuracy of the analysis, and the decision to submit for publication. The study was conceived by NM, CB and SU; data were prepared by KO and SG; the analysis was conducted by NL and SG; figures were prepared by NL; the paper was written by NM; all authors reviewed and contributed to the final draft submitted; the guarantor is NM.

Transparency declaration

The lead author NM affirms that the manuscript is an honest, accurate, and transparent account of the study being reported; that no important aspects of the study have been omitted.

The Corresponding Author has the right to grant on behalf of all authors and does grant on behalf of all authors, an exclusive licence on a worldwide basis to the BMJ Publishing Group Ltd to permit this article to be published in BMJ editions and any other BMJPGL products and sub-licences such use and exploit all subsidiary rights, as set out in the BMJ licence.

Funding source

We acknowledge funding for SFG through a Medical Research Council award and for NL through an award from the Health Data Research UK Hub Discover-NOW, both held by NM. The funders had no role in study design, collection, analysis, and interpretation of data, writing of the report, or the decision to submit the paper for publication.

Competing interest statement

All authors have completed the Unified Competing Interest form and declare no support from any organisation for the submitted work, no financial relationships with any organisations that might have an interest in the submitted work in the previous three years, no other relationships or activities that could appear to have influenced the submitted work. NM reports grants outside the submitted work from the Medical Research Council, National Institute of Health Research, March of Dimes, British Heart Foundation, HCA International, Health Data Research UK, Shire Pharmaceuticals, Chiesi Pharmaceuticals, Prolacta Life Sciences, and Westminster Children's Research Fund; NM is a member of the Nestle Scientific Advisory Board and accepts no personal remuneration for this role. NM reports travel and accommodation reimbursements from Chiesi, Nestle and Shire. NM is the

For peer review only - http://bmjopen.bmj.com/site/about/guidelines.xhtml

Chief Investigator for the National Neonatal Research Database. All other authors report no declarations of interest.

Data sharing statement

The National Neonatal Research Database is a UK national data asset. Details of access procedures are available https://www.imperial.ac.uk/neonatal-data-analysis-unit/neonatal-data-analysis-unit/utilising-the-national-neonatal-research-database/

Patient and public involvement

The National Neonatal Research Database has been developed in collaboration with parents and former patients; it is overseen by a Steering Board that includes parent representatives.

References

1 https://www.instituteforgovernment.org.uk/sites/default/files/timeline-lockdown-web.pdf [Last accessed 04-05-21]

2 Jephcote C, Hansell AL, Adams K, Gulliver J. Changes in air quality during COVID-19 'lockdown' in the United Kingdom Environmental Pollution 2021; 272:116011

3 UK Office for National Statistics. Domestic abuse during the coronavirus (COVID-19) pandemic, England and Wales: November 2020 https://www.ons.gov.uk/peoplepopulationandcommunity/crimeandjustice/articles/domesticabuseduringthecoronaviruscovid19pandemicenglandandwales/november2020#main-points (last accessed 27-04-21)

4 Sidpra J, Abomeli D, Hameed B, Baker J, Mankad K. Rise in the incidence of abusive head trauma during the COVID-19 pandemic Arch Dis Child 2020; 106:e14

5 NHS England Feb 18th 2020 https://www.england.nhs.uk/coronavirus/wp-content/uploads/sites/52/2020/03/coronavirus-primary-care-briefing.pdf; last accessed 20-05-21

6 Berghella V, Boelig R, Roman A, Burd J, Anderson K Decreased incidence of preterm birth during coronavirus disease 2019 pandemic (Research Letter). Am J Obstet Gynecol MFM 2020; 2:100258

7 Philip RK, Purtill H, Reidy E et al. Unprecedented reduction in births of very low birthweight (VLBW) and extremely low birthweight (ELBW) infants during the COVID-19 lockdown in Ireland: a 'natural experiment' allowing analysis of data from the prior two decades. BMJ Glob Health. 2020; 5(9):e003075

8 Been JV, Burgos Ochoa L, Bertens LCM, Schoenmakers S, Steegers EAP, Reiss IKM. Impact of COVID-19 mitigation measures on the incidence of preterm birth: a national quasi-experimental study. Lancet Public Health 2020; 5: e604–11

9 Hedermann G, Hedley PL, Baekvad-Hansen M, et al. Danish premature birth rates during the COVID-19 lockdown Arch Dis Child Fetal Neonatal Ed (2020) published online Aug 9; https://doi.org/10.1136/archdischild-2020-319990

10 Matheson A, McGannon CJ, Malhotra A et al. Prematurity rates during the coronavirus disease 2019 (COVID-19) pandemic lockdown in Melbourne, Australia. Obstet Gynecol 2021; 137:405-7

For peer review only - http://bmjopen.bmj.com/site/about/guidelines.xhtml

11 Lemon L, Edwards RP, Simhan HN. What is driving the decreased incidence of preterm birth during the coronavirus disease 2019 pandemic? Am J Obstet Gynecol MFM 2021; 3:100330

12 Maeda Y, Nakamura M, Ninomiya H, Ogawa K, Sago H, Miyawak A. Trends in intensive neonatal care during the COVID-19 outbreak in Japan. Arch Dis Child Fetal Neonatal Ed 2021; 106:F327-9

13 Handley SC, Mullin AM, Elovitz MA et al. Changes in preterm birth phenotypes and stillbirth at 2 Philadelphia hospitals during the SARS-CoV-2 pandemic, March-June 2020 (Research Letter). JAMA 2021; 325(1):87-9

14 Goldenberg RL, Culhane JF, Iams JD, Romero R. Epidemiology and causes of preterm birth Lancet 2008; 371:75-84

15 Morrison JJ, Rennie JM, Milton PJ. Neonatal respiratory morbidity and mode of delivery at term: influence of timing of elective caesarean section. Br J Obstet Gynaecol 1995; 102:101-6

16 Modi N, Ashby D, Battersby C et al. Developing routinely recorded clinical data from electronic patient records as a national resource to improve neonatal health care: the Medicines for Neonates research programme NIHR Journals Library Southampton (UK) 2019 Sep

17 Battersby C, Statnikov Y, Santhakumaran S et al. The United Kingdom National Neonatal Research Database: A validation study. PloS one. 2018;13(8)

18 Office for National Statistics https://www.ons.gov.uk/peoplepopulationandcommunity/birthsdeathsandmarriages/livebirths/articles/provisionalbirthsinenglandandwales/2020; (Last accessed 04-05-21)

19 NHS Data Model and Dictionary https://datadictionary.nhs.uk/data_elements/pds_ethnic_category_code.html; (Last accessed 04-05-21)

20 Ministry of Housing, Communities and Local Government. The English Indices of Deprivation 2019 https://assets.publishing.service.gov.uk/government/uploads/system/uploads/attachment_data/file/853811/IoD2019_FAQ_v4.pdf; (Last accessed 04-05-21)

21 Casella G. An Introduction to Empirical Bayes Data Analysis. The American Statistician, May 1985, Vol. 39, No. 2 (May, 1985), pp. 83-87

22 Charlesworth A. Shock to the system: COVID-19's long-term impact on the NHS. The Health Foundation 28th May 2020; https://www.health.org.uk/news-and-comment/blogs/shock-to-the-system-covid-19s-long-term-impact-on-the-nhs (last accessed 27-04-21)

23 Morello F, Bima P, Ferreri E et al. After the first wave and beyond lockdown: long-lasting changes in emergency department visit number, characteristics, diagnoses, and hospital admissions Intern Emerg Med 2021;March 8:1-8 Online ahead of print

24 Kirby T Evidence mounts on the disproportionate effect of COVID-19 on ethnic minorities. Lancet Respiratory Medicine 2020; 8:548

For peer review only - http://bmjopen.bmj.com/site/about/guidelines.xhtml

25 Public Health England Beyond the data: Understanding the impact of COVID-19 on BAME groups https://assets.publishing.service.gov.uk/government/uploads/system/uploads/attachment_data/file/892376/COVID_stakeholder_engagement_synthesis_beyond_the_data.pdf (Last accessed 04-05-21)

26 British Association of Perinatal Medicine 2019. Perinatal management of extremely preterm birth https://www.bapm.org/resources/80-perinatal-management-of-extreme-preterm-birth-before-27-weeks-of-gestation-2019; (Last accessed 04-05-21)

27 Khalil A, von Dadelszen P, Draycott T, Ugwumadu A, O'Brien P, Magee L. Change in the incidence of stillbirth and preterm delivery during the COVID-19 pandemic; Research Letter. JAMA. 2020; 324:705-6

28 Weightman AL, Morgan HE, Shepherd MA, Kitcher H, Roberts C, Dunstan FD. Social inequality and infant health in the UK: systematic review and meta-analyses. BMJ Open 2012 Jun 14; 2(3):e000964

29 Goodman J, de Prudhoe K, Williams C of the Covid-19 Bereaved Families for Justice. UK COVID-19 public inquiry needed to learn lessons and save lives. The Lancet 2021; 397: 177-80

30 https://www.theguardian.com/society/2021/mar/25/patient-safety-watchdog-launches-investigation-into-nhs-111-advice-to-covid-patients; last accessed 20-05-21

31 https://www.gov.uk/government/speeches/pm-house-of-commons-statement-on-covid-12-may-2021; last accessed 14-05-21

32 Modi N, Hanson M Health of women and children is central to covid-19 recovery BMJ 2021; 373:n899

For peer review only - http://bmjopen.bmj.com/site/about/guidelines.xhtml

Table 1

Summary of *highly unusual* changes in admissions to neonatal units in England and Wales during April-June 2020 (spring), the first three months of national COVID-19 lockdown; we considered a change *highly unusual* if the difference (whether positive or negative) between this period and December 2019-February 2020 (winter) was greater than the corresponding differences for all seven preceding years, or was in the opposite direction to all previous differences regardless of magnitude.

The P value reflects the uncertainty in the comparison of the spring-winter 2019-2020 differences and spring-winter differences in the previous seven years; the table lists all results for which the P value is less than 0.05.

N=absolute number; %=percentage of infants admitted in gestational age category

For peer review only

Highly unusual changes	Gestational age category	Direction of change (Apr-Jun 2020 compared with Dec 2019-Feb 2020	Absolute magnitude of change (Apr-Jun 2020 compared with Dec 2019-Feb 2020	Range of change between Apr-Jun and preceding Dec-Feb in the years 2012-2019	P value
Total babies admitted (N)	• All preterm	Decrease	-350	-26, +479	<0.001
	• Full-term	Decrease	-1142	+104, +1178	<0.001
	• All admissions	Decrease	-1492	+100, +1617	<0.001
Black ethnicity (N)	• Full-term	Increase	+66	-64, +35	<0·001
Asian ethnicity (N)	• All admissions	Decrease	-137	-14, +101	<0.001
White ethnicity (N)	• Full-term	Decrease	-218	-21, +365	<0.001
	• All admissions	Decrease	-319	-235, +643	<0.001

Socio-economic quintile two	• Very preterm	Decrease	-41	-20, +59	0.036
Socio-economic quintile three	• Full-term	Decrease	-148	+28, +307	<0.001
Socio-economic quintile four	• Full-term	Decrease	-135	-39, +198	<0.001
Socio-economic quintile five (least deprived)	• Moderate to late preterm<br>• Full-term	Decrease<br>Decrease	-51<br>-175	-8, +58<br>+17, +164	<0.001<br><0.001
Elective Caesarean section (N)	• Very preterm	Decrease	-27	-17, +34	0.035
Elective Caesarean section (%)	• Very preterm	Decrease	-2.3%	-1.3, +2.0	0.035
Born in hospital with level 3 neonatal unit (intensive care) (N)	• Extremely preterm	Decrease	-40	+3, +71	0.027
Transfer to higher designation neonatal unit (N)	• Moderate-to-late preterm<br>• Full-term<br>• All admissions	Increase<br>Increase<br>Increase	+37<br>+69<br>+129	-8, +18<br>+10, +53<br>-4, +88	0.007<br><0.001<br><0.001
Transfer to lower designation neonatal unit (N)	• Full-term<br>• All admissions	Decrease<br>Decrease	-15<br>-47	-8, +3<br>-25, +12	0.004<br><0.001
Necrotising enterocolitis surgery (%)	• Very preterm	Decrease	-1.1%	-0.9%, +0.1%	0.017

For peer review only - http://bmjopen.bmj.com/site/about/guidelines.xhtml

Breast-feeding at discharge (N)	• Moderate-to-late preterm<br>• Full-term	Decrease<br>Decrease	-202<br>-65	-91, +170<br>-38, +267	0.031<br>0.015
Breast-feeding at discharge (%)	• Moderate-to-late preterm<br>• Full-term	Decrease<br>Increase	-1.7%<br>+1.4%	-1.1%, +1.5%<br>-1.2%, +1.0%	0.047<br>0.031

Table 2 Numbers of mothers and babies with suspected and confirmed SARS-CoV-2 infection

	Mother		Baby	
	Suspected	Confirmed	Suspected	Confirmed
Dec 2019 - Feb 2020	22	9	46	8
Apr 2020 - Jun 2020	486	89	139	13
Jul 2020 - Sep 2020	189	42	20	3

For peer review only - http://bmjopen.bmj.com/site/about/guidelines.xhtml

Figure legends

Figure 1

Admissions to neonatal units in England and Wales by gestational age category, and year

GA1: Extremely preterm; GA2: very preterm; GA3: moderate-to-late preterm; GA4: full-term; black circle: December to February; black square: April to June; grey triangle: July to September. The COVID period is highlighted; the thick black lines indicate a change that was highly unusual.

There was a highly unusual fall in all preterm (GA groups 1-3 combined) and full-term (GA4) admissions during the period April to June 2020. The falls in GA1 and GA3 admissions were individually also highly unusual; the falls in GA1 and GA2, the most immature babies, continued into the period July to September 2020, unlike GA3 and GA4 which rose again.

Figure 2

Admissions to neonatal units in England and Wales by gestational age category, year and Index of Multiple Deprivation (IMD) quintile

Black circle: December to February; black square: April to June; grey triangle: July to September. The COVID period is shaded; the thick black lines indicate a change that was highly unusual; GA1: Extremely preterm; GA2: very preterm; GA3: moderate-to-late preterm; GA4: full-term; Q1: quintile 1 (most deprived); Q5: least deprived)

There were highly unusual falls in GA1 (extremely preterm) admissions in IMD quintiles 1 and 2, and in GA2 (very preterm) admissions in IMD quintile 2 over April-June 2020; the fall in GA1 (extremely preterm) admissions was sustained into the period July to September. In contrast, there was a highly unusual fall in GA 3 (moderate-to-late preterm) admissions over the COVID period only in IMD quintile 5 and in GA4 (full-term) admissions in quintiles 2, 3, 4 and 5.

Figure 3

Admissions of Black ethnicity babies to neonatal units in England and Wales by year and period

GA1: Extremely preterm; GA2: very preterm; GA3: moderate-to-late preterm; GA4: full-term; black circle: December to February; black square: April to June; grey triangle: July to September. The COVID period is highlighted; the thick black lines indicate a change that was highly unusual.

There was a highly unusual increase in all admissions (GA groups 1-4 combined) over April to June 2020, driven by the full-term (GA4) category. This increase was not sustained into the period July to September 2020

Figure 4

Live births and stillbirths births, England and Wales by 2013 to 2020 and period

Black circle: December to February; black square: April to June; grey triangle: July to September; The COVID period is highlighted.

For peer review only

For peer review only - http://bmjopen.bmj.com/site/about/guidelines.xhtml

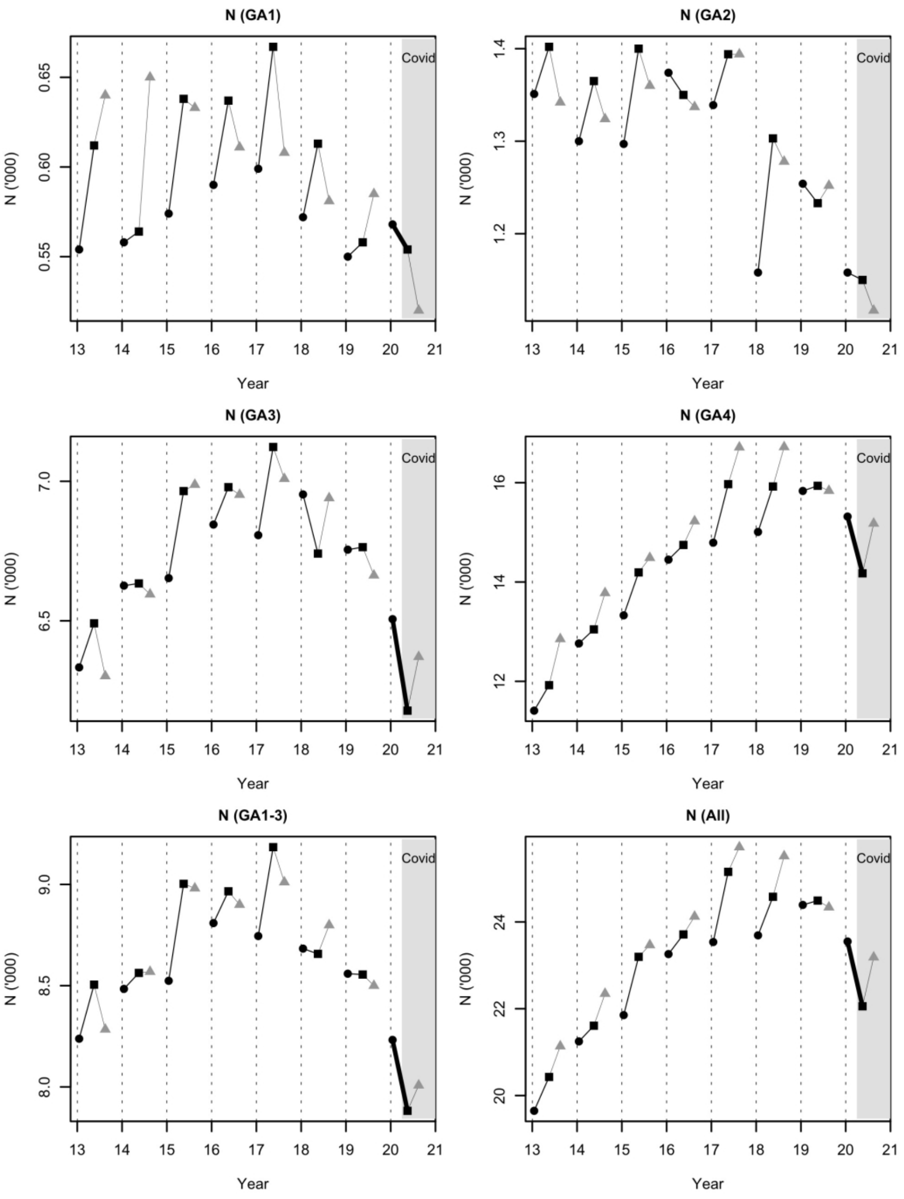

Figure 1
Admissions to neonatal units in England and Wales by gestational age category, and year
GA1: Extremely preterm; GA2: very preterm; GA3: moderate-to-late preterm; GA4: full-term; black circle: December to February; black square: April to June; grey triangle: July to September. The COVID period is highlighted; the thick black lines indicate a change that was highly unusual.
There was a highly unusual fall in all preterm (GA groups 1-3 combined) and full-term (GA4) admissions during the period April to June 2020. The falls in GA1 and GA3 admissions were individually also highly unusual; the falls in GA1 and GA2, the most immature babies, continued into the period July to September 2020, unlike GA3 and GA4 which rose again.

698x920mm (72 x 72 DPI)

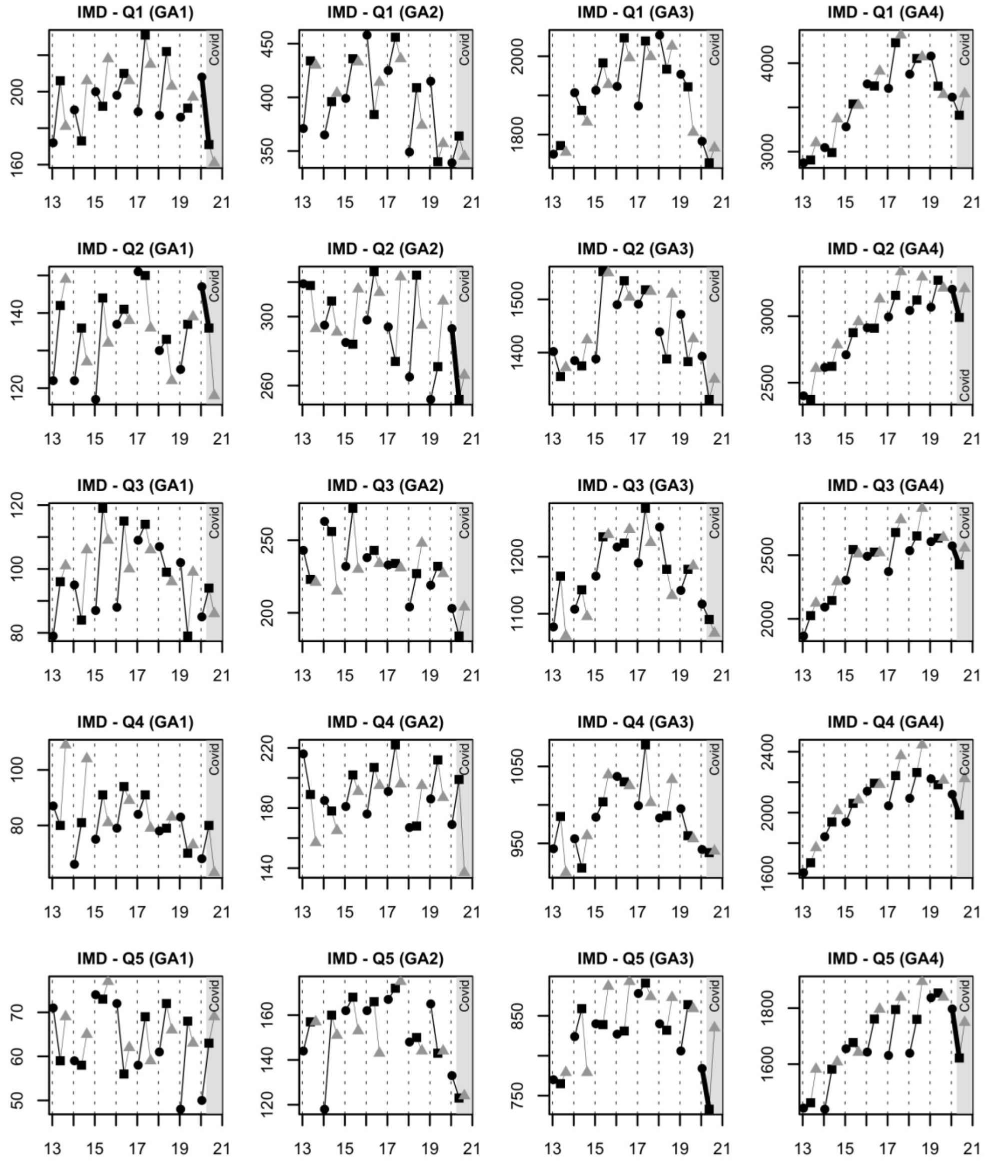

Figure 2
Admissions to neonatal units in England and Wales by gestational age category, year and Index of Multiple Deprivation (IMD) quintile
Black circle: December to February; black square: April to June; grey triangle: July to September. The COVID period is shaded; the thick black lines indicate a change that was highly unusual; GA1: Extremely preterm; GA2: very preterm; GA3: moderate-to-late preterm; GA4: full-term; Q1: quintile 1 (most deprived); Q5: least deprived)
There were highly unusual falls in GA1 (extremely preterm) admissions in IMD quintiles 1 and 2, and in GA2 (very preterm) admissions in IMD quintile 2 over April-June 2020; the fall in GA1 (extremely preterm) admissions was sustained into the period July to September. In contrast, there was a highly unusual fall in GA 3 (moderate-to-late preterm) admissions over the COVID period only in IMD quintile 5 and in GA4 (full-term) admissions in quintiles 2, 3, 4 and 5.

698x825mm (72 x 72 DPI)

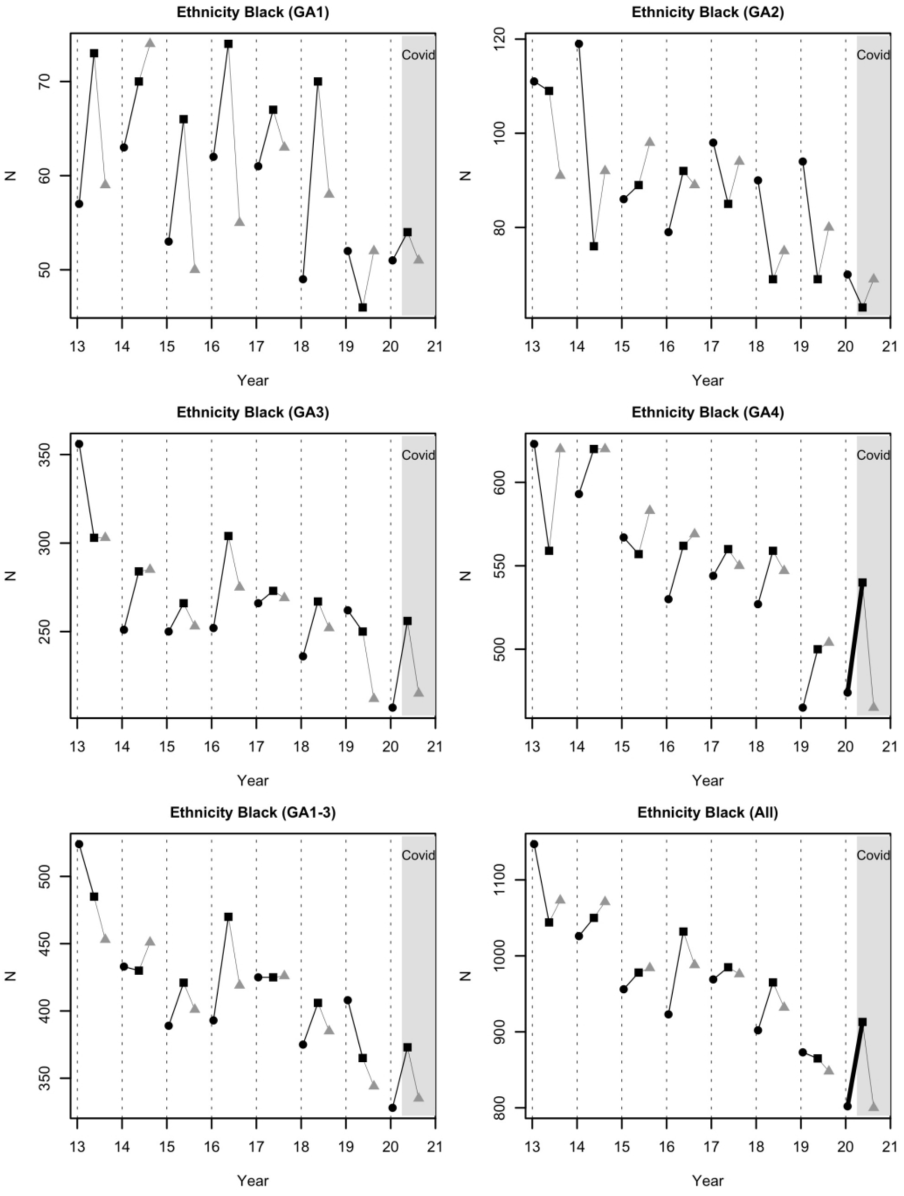

Figure 3

Admissions of Black ethnicity babies to neonatal units in England and Wales by year and period

GA1: Extremely preterm; GA2: very preterm; GA3: moderate-to-late preterm; GA4: full-term; black circle: December to February; black square: April to June; grey triangle: July to September. The COVID period is highlighted; the thick black lines indicate a change that was highly unusual.

There was a highly unusual increase in all admissions (GA groups 1-4 combined) over April to June 2020, driven by the full-term (GA4) category. This increase was not sustained into the period July to September 2020

698x920mm (72 x 72 DPI)

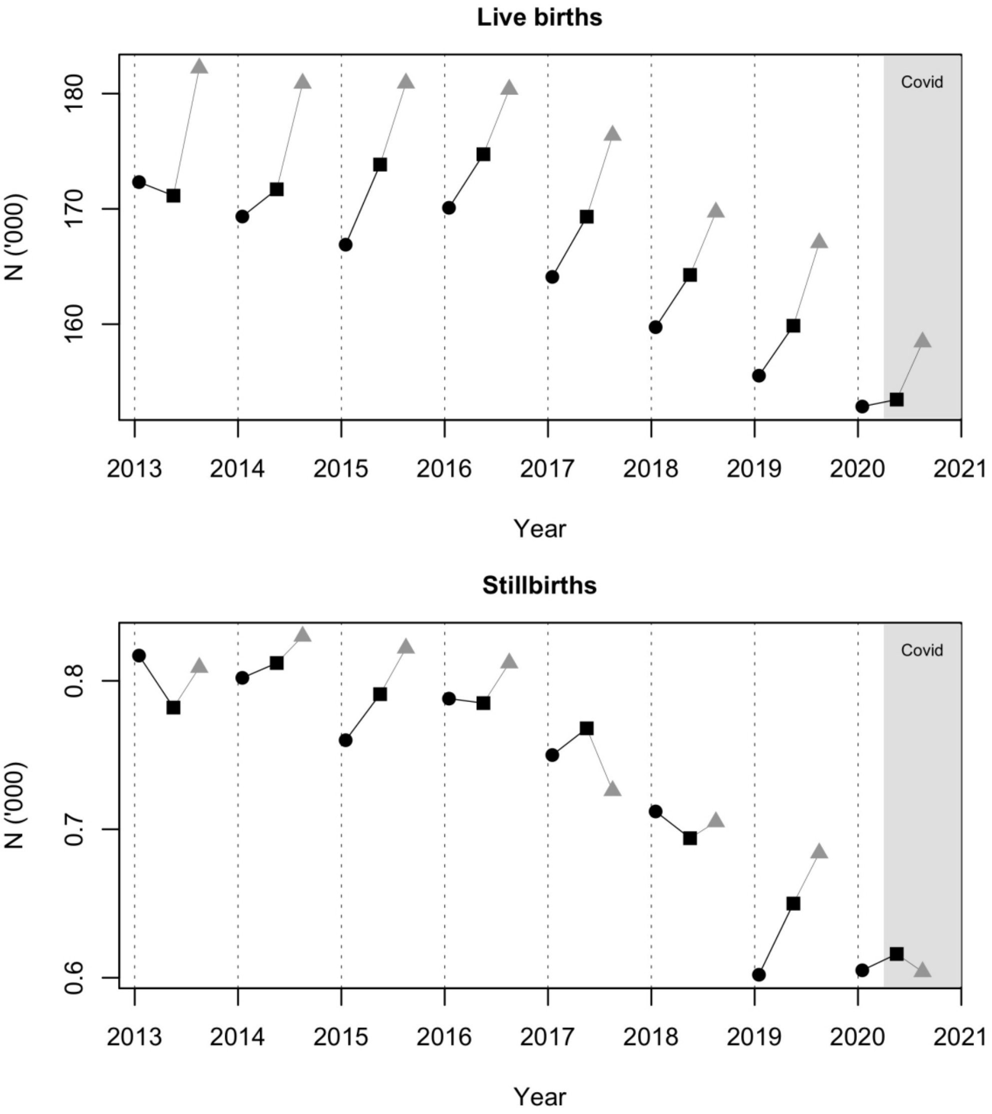

Figure 4
Live births and stillbirths births, England and Wales by 2013 to 2020 and period
Black circle: December to February; black square: April to June; grey triangle: July to September; The COVID period is highlighted.

698x793mm (72 x 72 DPI)

Supplementary Table S1

UK Neonatal Collaborative hospitals and lead clinicians in England and Wales

**Hospital**	**Lead clinician**
Airedale General	Dr Matthew Babirecki
Arrowe Park	Dr Anand Kamalanathan
Barnet	Dr Tim Wickham
Barnsley District General	Dr Kavi Aucharaz
Basildon	Dr Aashish Gupta
Basingstoke & North Hampshire	Dr Nicola Paul
Bassetlaw District General	Dr L M Wong
Bedford	Dr Anita Mittal
Birmingham City	Dr Penny Broggio
Birmingham Heartlands	Dr Pinki Surana
Birmingham Women's	Dr Matt Nash
Bradford Royal Infirmary	Dr Sunita Seal
Broomfield, Chelmsford	Dr Ahmed Hassan
Calderdale Royal Hospital	Dr Karin Schwarz
Chelsea & Westminster	Dr Shu-Ling Chuang
Chesterfield & North Derbyshire Royal	Dr Aiwyne Foo
Colchester General	Dr Jo Anderson
Conquest	Dr Graham Whincup
Countess of Chester	Dr Stephen Brearey
Croydon University	Dr John Chang
Cumberland Infirmary	Dr Yee Aung
Darent Valley	Dr Abdul Hasib
Darlington Memorial Hospital	Dr Mehdi Garbash
Derriford Hospital	Dr Alex Allwood
Diana Princess of Wales	Dr Pauline Adiotomre
Doncaster Royal Infirmary	Dr Nigel Brooke
Dorset County	Dr Abby Deketelaere
East Surrey	Dr K Abdul Khader
Epsom General	Dr Ruth Shephard
Frimley Park	Dr Sanghavi Rekha
Furness General	Dr Anas Olabi
George Eliot	Dr Mukta Jain
Gloucester Royal	Dr Jennifer Holman
Good Hope	Dr Pinki Surana
Great Western	Dr Stanley Zengeya
Guy's & St Thomas'	Dr Geraint Lee
Harrogate District	Dr Sobia Balal
Hereford County	Dr Cath Seagrave
Hillingdon	Dr Tristan Bate
Hinchingbrooke	Dr Hilary Dixon
Homerton	Dr Narendra Aladangady
Hull Royal Infirmary	Dr Hassan Gaili
Ipswich	Dr Matthew James
James Cook University	Dr M Lal

For peer review only

James Paget	Dr Ambadkar
Kettering General	Dr Poornima Pandey
Kings College	Dr Ravindra Bhat
King's Mill	Dr Simon Rhodes
Kingston	Dr Vinay Pai
Lancashire Women and Newborn Centre	Dr Savi Sivashankar
Leeds	Dr Lawrence Miall
Leicester General	Dr Jonathan Cusack
Leicester Royal Infirmary	Dr Venkatesh Kairamkonda
Leighton	Dr Michael Grosdenier
Lincoln County	Dr Kollipara
Lister	Dr J Kefas
Liverpool Women's	Dr Christopher Dewhurst
Luton & Dunstable	Dr Jennifer Birch
Macclesfield District General	Dr Gail Whitehead
Manor	Dr Krishnamurthy
Medway Maritime	Dr Ghada Ramadan
Milton Keynes General	Dr I Misra
Musgrove Park	Dr Chris Knight
New Cross	Dr Tilly Pillay
Newham General	Dr Imdad Ali
Nobles	Dr Prakash Thiagarajan
Norfolk & Norwich University	Dr Mark Dyke
North Devon District	Dr Michael Selter
North Manchester General	Dr P Kamath
North Middlesex University	Dr Neeraj Jain
Northumbria Specialist Emergency Care	Vivien Spencer
Northampton General	Dr Subodh Gupta
Northwick Park	Dr Richard Nicholl
Nottingham City	Dr Steven Wardle
Nottingham University	Dr Steven Wardle
Ormskirk District General	Dr Andreea Bontea
John Radcliffe	Dr Eleri Adams
Peterborough City	Dr Katharine McDevitt
Pilgrim	Dr Ajay Reddy
Pinderfields General (Pontefract General Infirmary)	Dr David Gibson
Poole General	Prof Minesh Khashu
Princess Alexandra	Dr Chinnappa Reddy
Princess Anne	Dr Mark Johnson
Princess Royal	Dr P Amess
Princess Royal (previously Royal Shrewsbury)	Dr Deshpande
Princess Royal University	Dr Elizabeth Sleight
Queen Alexandra	Dr Charlotte Groves
Queen Charlotte's	Dr Lidia Tyszczuk
Queen Elizabeth, Gateshead	Dr Dennis Bosman
Queen Elizabeth, King's Lynn	Dr Glynis Rewitzky
Queen Elizabethl, Woolwich	Dr Olutoyin Banjoko
Queen Elizabeth the Queen Mother	Dr Bushra Abdul-Malik

For peer review only

Queen's Hospital, Burton on Trent	Dr Dominic Muogbo
Queen's Hospital, Romford	Dr Khalid Mannan
Queen's Hospital, Romford 2	Dr Anand Shirsalkar
Rosie Maternity, Addenbrookes	Dr Angela D'Amore
Rotherham District General	Dr Shameel Mattara
Royal Albert Edward Infirmary	Dr Christos Zipitis
Royal Berkshire	Dr Peter De Halpert
Royal Bolton	Dr Paul Settle
Royal Cornwall	Dr Paul Munyard
Royal Derby	Dr John McIntyre
Royal Devon & Exeter	Dr David Bartle
Royal Hampshire County	Dr Lucinda Winckworth
Royal Lancaster Infirmary	Dr Joanne Fedee
Royal Oldham	Dr Natasha Maddock
Royal Preston	Dr Richa Gupta
Royal Stoke University	Dr Alison Moore
Royal Surrey County	Dr Ben Obi
Royal Sussex County	Dr Phil Amess
Royal United Hospital	Dr Stephen Jones
Royal Victoria Infirmary	Dr Naveen Athiraman
Russells Hall	Dr Mahadevan
Salisbury District	Dr Jim Baird
Scarborough General	Dr Kirsten Mack
Scunthorpe General	Dr Pauline Adiotomre
Southend	Dr Vineet Gupta
Southmead	Dr Alison Pike
St George's	Dr Charlotte Huddy
St Helier	Dr Salim Yasin
St Mary's, Isle of Wight	Dr Sian Butterworth
St Mary's, London	Dr Lidia Tyszczuk
St Mary's, Manchester	Dr Ngozi Edi-Osagie
St Michael's	Dr Pamela Cairns
St Peter's	Dr Peter Reynolds
St Richard's	Dr Nick Brennan
Stepping Hill	Dr Carrie Heal
Stoke Mandeville	Dr Sanjay Salgia
Sunderland Royal	Dr Majd Abu-Harb
Tameside General	Dr Jacqeline Birch
The Jessop Wing, Sheffield	Dr Porus Bastani
The Royal Free	Dr Marice Theron
The Royal London	Dr Vadivelam Murthy
Torbay	Dr Siba Paul
Tunbridge Wells	Dr Hamudi Kisat
University College	Dr Giles Kendall
University Hospital Coventry	Dr Kate Blake
University Hospital Lewisham	Dr Ozioma Obi
University Hospital of North Durham	Dr Mehdi Garbash
University Hospital of North Tees	Dr Hari Kumar
Victoria Hospital, Blackpool	Dr Chris Rawlingson

For peer review only

Warrington	Dr Delyth Webb
Warwick	Dr Bird
Watford General	Dr Sankara Narayanan
West Cumberland	no lead
West Middlesex University	Dr Eleanor Hulse
West Suffolk	Dr Ian Evans
Wexham Park	Dr Rekha Sanghavi
Whipps Cross University	Dr Caroline Sullivan
Whiston	Dr Ros Garr
Whittington	Dr Wynne Leith
William Harvey	Dr Vimal Vasu
Worcestershire Royal	Dr Liza Harry
Worthing	Dr Katia Vamvakiti
Wythenshawe	Dr Ngozi Edi-Osagie
Yeovil District	Dr Megan Eaton
York District	Dr Sundeep Sandhu

Wales

Hospital	Lead clinician
Singleton	Dr Arun Ramachandran
Princess of Wales	Dr Kate Creese
Royal Gwent	Dr Sunil Reddy
Nevill Hall Hospital	Dr Sunil Reddy
Glan Clwyd l	Dr Ian Barnard
Wrexham Maelor	Dr Brendan Harrington
Ysbyty Gwynedd	Dr Mike Cronin
University Hospital of Wales	Dr Alok Sharma
Prince Charles	Dr Iyad Al-Muzaffar
Glangwili General	Dr Prem Pitchaikani
Withybush	Dr Vishwa Narayan

For peer review only

The RECORD statement – checklist of items, extended from the STROBE statement, that should be reported in observational studies using routinely collected health data.

	Item No.	STROBE items	Location in manuscript where items are reported	RECORD items	Location in manuscript where items are reported
**Title and abstract**					
	1	(a) Indicate the study's design with a commonly used term in the title or the abstract (b) Provide in the abstract an informative and balanced summary of what was done and what was found		RECORD 1.1: The type of data used should be specified in the title or abstract. When possible, the name of the databases used should be included.<br><br>RECORD 1.2: If applicable, the geographic region and timeframe within which the study took place should be reported in the title or abstract.<br><br>RECORD 1.3: If linkage between databases was conducted for the study, this should be clearly stated in the title or abstract.	Abstract; page 2<br><br>Abstract; page 2<br><br>Not applicable; no linkage between databases
**Introduction**					
Background rationale	2	Explain the scientific background and rationale for the investigation being reported			Section headed "Introduction"; pages 4-6
Objectives	3	State specific objectives, including any prespecified hypotheses			Section headed "Introduction"; page 4
**Methods**					
Study Design	4	Present key elements of study design early in the paper			Sections headed "Introduction" (page 4)
Setting	5	Describe the setting, locations, and relevant dates, including periods of recruitment, exposure, follow-up, and data collection			Sections headed "Introduction" (page 4), "Methods" (pages

For peer review only

					4); "Analyses" (pages 5-6)
Participants	6	*(a) Cohort study* - Give the eligibility criteria, and the sources and methods of selection of participants. Describe methods of follow-up<br>*Case-control study* - Give the eligibility criteria, and the sources and methods of case ascertainment and control selection. Give the rationale for the choice of cases and controls<br>*Cross-sectional study* - Give the eligibility criteria, and the sources and methods of selection of participants<br><br>*(b) Cohort study* - For matched studies, give matching criteria and number of exposed and unexposed<br>*Case-control study* - For matched studies, give matching criteria and the number of controls per case		RECORD 6.1: The methods of study population selection (such as codes or algorithms used to identify subjects) should be listed in detail. If this is not possible, an explanation should be provided.<br><br>RECORD 6.2: Any validation studies of the codes or algorithms used to select the population should be referenced. If validation was conducted for this study and not published elsewhere, detailed methods and results should be provided.<br><br>RECORD 6.3: If the study involved linkage of databases, consider use of a flow diagram or other graphical display to demonstrate the data linkage process, including the number of individuals with linked data at each stage.	Section headed "Neonatal admissions" (page 5)<br><br>References 16-18<br><br>No linkage of databases
Variables	7	Clearly define all outcomes, exposures, predictors, potential confounders, and effect modifiers. Give diagnostic criteria, if applicable.		RECORD 7.1: A complete list of codes and algorithms used to classify exposures, outcomes, confounders, and effect modifiers should be provided. If these cannot be reported, an explanation should be provided.	Section headed "Outcomes" (page 5)
Data sources/ measurement	8	For each variable of interest, give sources of data and details of methods of assessment (measurement).			Section headed "Data sources" (pages 4-5)

For peer review only

		Describe comparability of assessment methods if there is more than one group			
Bias	9	Describe any efforts to address potential sources of bias			Section headed "Analyses" (pages 5-6)
Study size	10	Explain how the study size was arrived at			Section headed "Neonatal Admissions" (page 5)
Quantitative variables	11	Explain how quantitative variables were handled in the analyses. If applicable, describe which groupings were chosen, and why			Section headed "Outcomes" (page 5)
Statistical methods	12	(a) Describe all statistical methods, including those used to control for confounding<br>(b) Describe any methods used to examine subgroups and interactions<br>(c) Explain how missing data were addressed<br>(d) *Cohort study* - If applicable, explain how loss to follow-up was addressed<br>*Case-control study* - If applicable, explain how matching of cases and controls was addressed<br>*Cross-sectional study* - If applicable, describe analytical methods taking account of sampling strategy<br>(e) Describe any sensitivity analyses			Section headed "Analyses" (pages 5-6)

For peer review only

Data access and cleaning methods		..		RECORD 12.1: Authors should describe the extent to which the investigators had access to the database population used to create the study population.<br><br>RECORD 12.2: Authors should provide information on the data cleaning methods used in the study.	Section headed "Contributor Statement" (page 10)<br><br>Section headed "Neonatal admissions" (page 5)
Linkage		..		RECORD 12.3: State whether the study included person-level, institutional-level, or other data linkage across two or more databases. The methods of linkage and methods of linkage quality evaluation should be provided.	Not applicable
**Results**					
Participants	13	(a) Report the numbers of individuals at each stage of the study (*e.g.*, numbers potentially eligible, examined for eligibility, confirmed eligible, included in the study, completing follow-up, and analysed)<br>(b) Give reasons for non-participation at each stage.<br>(c) Consider use of a flow diagram		RECORD 13.1: Describe in detail the selection of the persons included in the study (*i.e.,* study population selection) including filtering based on data quality, data availability and linkage. The selection of included persons can be described in the text and/or by means of the study flow diagram.	Section headed "Results" (page 6)
Descriptive data	14	(a) Give characteristics of study participants (*e.g.*, demographic, clinical, social) and information on exposures and potential confounders<br>(b) Indicate the number of participants with missing data for each variable of interest			Section headed "Results" (page 6)

For peer review only

		(c) *Cohort study* - summarise follow-up time (*e.g.*, average and total amount)			
Outcome data	15	*Cohort study* - Report numbers of outcome events or summary measures over time<br>*Case-control study* - Report numbers in each exposure category, or summary measures of exposure<br>*Cross-sectional study* - Report numbers of outcome events or summary measures			Section headed "Results" (page 6)
Main results	16	(a) Give unadjusted estimates and, if applicable, confounder-adjusted estimates and their precision (e.g., 95% confidence interval). Make clear which confounders were adjusted for and why they were included<br>(b) Report category boundaries when continuous variables were categorized<br>(c) If relevant, consider translating estimates of relative risk into absolute risk for a meaningful time period			Section headed "Results" (page 6)
Other analyses	17	Report other analyses done—e.g., analyses of subgroups and interactions, and sensitivity analyses			Section headed "Results" (page 6)
**Discussion**					
Key results	18	Summarise key results with reference to study objectives			Section headed "Results" (page 6)
Limitations	19	Discuss limitations of the study, taking into account sources of potential bias or imprecision.		RECORD 19.1: Discuss the implications of using data that were not created or collected to answer the specific research question(s). Include	Paragraph 5 of section headed "Discussion" (page 7)

For peer review only

		Discuss both direction and magnitude of any potential bias		discussion of misclassification bias, unmeasured confounding, missing data, and changing eligibility over time, as they pertain to the study being reported.	
Interpretation	20	Give a cautious overall interpretation of results considering objectives, limitations, multiplicity of analyses, results from similar studies, and other relevant evidence			Sections headed "Discussion" (page 7)
Generalisability	21	Discuss the generalisability (external validity) of the study results			Sections headed "Discussion" (page 7)
**Other Information**					
Funding	22	Give the source of funding and the role of the funders for the present study and, if applicable, for the original study on which the present article is based			Section headed "Funding Source" (page 10)
Accessibility of protocol, raw data, and programming code		..		RECORD 22.1: Authors should provide information on how to access any supplemental information such as the study protocol, raw data, or programming code.	Sections headed "Data Sharing Statement" (page 11)

*Reference: Benchimol EI, Smeeth L, Guttmann A, Harron K, Moher D, Petersen I, Sørensen HT, von Elm E, Langan SM, the RECORD Working Committee. The REporting of studies Conducted using Observational Routinely-collected health Data (RECORD) Statement. *PLoS Medicine* 2015; in press.

*Checklist is protected under Creative Commons Attribution (CC BY) license.

For peer review only

BMJ Open

Changes in neonatal admissions, care processes and outcomes in England and Wales during the COVID-19 pandemic: a whole population cohort study

Journal:	*BMJ Open*
Manuscript ID	bmjopen-2021-054410.R1
Article Type:	Original research
Date Submitted by the Author:	05-Sep-2021
Complete List of Authors:	Greenbury, Sam; Imperial College London, Section of Neonatal Medicine, School of Public Health, Faculty of Medicine; Imperial College London, Institute for Translational Medicine and Therapeutics Data Science Group<br>Longford, Nicholas; Imperial College London, Section of Neonatal Medicine<br>Ougham, Kayleigh; Imperial College London, Section of Neonatal Medicine, School of Public Health, Faculty of Medicine<br>Angelini, Elsa; Imperial College London, Institute for Translational Medicine and Therapeutics Data Science Group<br>Battersby, Cheryl; Imperial College London, NHS England<br>Uthaya, Sabita; Imperial College London, Section of Neonatal Medicine, School of Public Health, Faculty of Medicine<br>Modi, Neena; Imperial College London, Medicine
**Primary Subject Heading**:	Paediatrics
Secondary Subject Heading:	Public health
Keywords:	Neonatal intensive & critical care < INTENSIVE & CRITICAL CARE, NEONATOLOGY, PUBLIC HEALTH

SCHOLARONE™
Manuscripts

I, the Submitting Author has the right to grant and does grant on behalf of all authors of the Work (as defined in the below author licence), an exclusive licence and/or a non-exclusive licence for contributions from authors who are: i) UK Crown employees; ii) where BMJ has agreed a CC-BY licence shall apply, and/or iii) in accordance with the terms applicable for US Federal Government officers or employees acting as part of their official duties; on a worldwide, perpetual, irrevocable, royalty-free basis to BMJ Publishing Group Ltd ("BMJ") its licensees and where the relevant Journal is co-owned by BMJ to the co-owners of the Journal, to publish the Work in this journal and any other BMJ products and to exploit all rights, as set out in our licence.

The Submitting Author accepts and understands that any supply made under these terms is made by BMJ to the Submitting Author unless you are acting as an employee on behalf of your employer or a postgraduate student of an affiliated institution which is paying any applicable article publishing charge ("APC") for Open Access articles. Where the Submitting Author wishes to make the Work available on an Open Access basis (and intends to pay the relevant APC), the terms of reuse of such Open Access shall be governed by a Creative Commons licence – details of these licences and which Creative Commons licence will apply to this Work are set out in our licence referred to above.

Other than as permitted in any relevant BMJ Author's Self Archiving Policies, I confirm this Work has not been accepted for publication elsewhere, is not being considered for publication elsewhere and does not duplicate material already published. I confirm all authors consent to publication of this Work and authorise the granting of this licence.

For peer review only

Changes in neonatal admissions, care processes and outcomes in England and Wales during the COVID-19 pandemic: a whole population cohort study

Sam F Greenbury PhD [1, 2], Nicholas T Longford PhD [1], Kayleigh Ougham MSc [1], Elsa D Angelini PhD [2], Cheryl Battersby FRCPH [1], Sabita Uthaya FRCP [1], Neena Modi FMedSci [1]

1 Section of Neonatal Medicine, School of Public Health, Faculty of Medicine, Chelsea and Westminster Hospital campus, Imperial College London, London, UK

2 National Institute for Health Research Imperial Biomedical Research Centre, Institute for Translational Medicine and Therapeutics Data Science Group, Imperial College London, London, UK

Correspondence to Professor N Modi, Section of Neonatal Medicine, School of Public Health, Faculty of Medicine, Imperial College London, Chelsea and Westminster Hospital campus, 369 Fulham Road, London SW10 9NH, UK; email: n.modi@imperial.ac.uk; tel: +44 (0) 203 315 5101

Manuscript word count: 3786

Keywords: neonatal; admissions; COVID-19; births; preterm; full-term; pandemic; healthcare; outcomes; National Neonatal Research Database

For peer review only - http://bmjopen.bmj.com/site/about/guidelines.xhtml

Abstract

300w

Objectives: The COVID-19 pandemic instigated multiple societal and healthcare interventions with potential to affect perinatal practice. We evaluated population-level changes in preterm and full-term admissions to neonatal units, care processes, and outcomes.

Design: Observational cohort study utilising the UK National Neonatal Research Database

Setting: England and Wales

Participants: Admissions to National Health Service neonatal units from 2012-2020

Main outcome measures: Admissions by gestational age, ethnicity and Index of Multiple Deprivation, and key care processes and outcomes

Methods: We calculated differences in numbers and rates between April-June 2020 (spring) the first three months of national lockdown (COVID period), and December 2019-February 2020 (winter), prior to introduction of mitigation measures, and compared them with the corresponding differences in the seven previous years. We considered the COVID period highly unusual if the spring-winter difference was smaller or larger than all previous corresponding differences, and calculated the level of confidence in this conclusion.

Results: Marked fluctuations occurred in all measures over the eight years with several highly unusual changes during the COVID period. Total admissions fell, having risen over all previous years (COVID difference: -1492; previous seven-year difference range: +100, +1617; p<0.001); full-term Black admissions rose (+66; -64, +35; p<0.001) whereas Asian (-137; -14, +101; p<0.001) and White (-319; -235, +643: p<0.001) admissions fell. Transfers to higher and lower designation neonatal units increased (+129; -4, +88; p<0.001) and decreased (-47; -25, +12; p<0.001), respectively. Total preterm admissions decreased (-350; -26, +479; p<0.001). The fall in extremely preterm admissions was most marked in the two lowest socio-economic quintiles.

Conclusions: Our findings indicate substantial changes occurred in care pathways and clinical thresholds, with disproportionate effects on Black ethnic groups, during the immediate COVID-19 period, and raise the intriguing possibility that non-healthcare interventions may reduce extremely preterm births.

For peer review only - http://bmjopen.bmj.com/site/about/guidelines.xhtml

Strengths and limitations of this study

- Our study is a complete population evaluation that included all admissions to NHS neonatal units in England and Wales over an eight-year period
- We assessed full-term, as well as extremely preterm, very preterm, and moderate-to-late preterm groups individually
- All previous studies have compared a COVID period with earlier periods with the implicit assumption that COVID-19 is the only agent likely to have influenced outcomes; however we show clearly there have been marked fluctuations in outcomes over time, hence assessed differences between the first national COVID-19 lockdown period and the preceding quarter, and compared these with corresponding differences in the previous seven years
- A limitation of our approach is that our measure of exceptionality may be too conservative, potentially hindering detection of a COVID-19 effect
- We were unable to evaluate national data on births by gestational age directly as these were not available

For peer review only

For peer review only - http://bmjopen.bmj.com/site/about/guidelines.xhtml

Introduction

The COVID-19 pandemic, the consequence of the emergence of a novel virus, SARS-CoV-2, has had potential to affect maternal and newborn health in multiple ways. In the United Kingdom (UK), the first full national lockdown commenced on March 23rd 2020 (1). This included requiring people to stay at home except for essential reasons, closure of public venues and all non-essential businesses, and prohibition of public gatherings. The national lockdown, and other policies implemented in an attempt to mitigate the spread of the virus, led to changes in hospital and general practitioner care, and alterations in environmental and societal factors. Thus, air quality improved in many highly populated urban areas (2), but reports of mental stress, domestic violence and child abuse, increased (3, 4). On February 18th 2020, NHS England advised the UK public not to contact their general practitioners, or go to hospital Accident and Emergency Departments, but instead to contact the NHS111 online and telephone service for medical advice (5). Within hospitals, In addition to the direct consequences of infection, the abrupt onset of the pandemic necessitated rapid implementation of changes in healthcare processes based on standard infection-control policies, without specific knowledge of the transmissibility, pathogenicity and epidemiology of the novel virus. The rapidity of spread led to re-deployment of healthcare staff and prioritised allocation of resources, such as personal protective equipment, to areas of greatest need.

There have been eight reports evaluating preterm births in relation to the onset of the pandemic; seven describe a reduction (5-12), and one no change (13). The spontaneous onset of preterm labour is associated with a number of factors, including infection, systemic illness, severe stress, and physical injury. From an epidemiological perspective, seasonal effects, socio-economic factors and population characteristics also affect the preterm birth rate (14). The pandemic might have additionally have influenced rates of elective Caesarean section, with and without medical indication, which are an iatrogenic cause of late preterm births, and a well-recognised cause of respiratory and other problems that lead to neonatal unit admission (15). However, the incidence of births by elective Caesarean section varies by population demographics, across healthcare systems and with time. Thus, for many reasons, identifying any causal determinants of preterm birth is problematic.

Our aim in this study was to determine if any "highly unusual" changes in admissions to neonatal units in England and Wales, care processes and outcomes occurred following the start of the first national lockdown. Recognising the marked fluctuations in these measures over time, we determined if changes in the immediate COVID-19 period, namely April to June 2020, when compared with the preceding quarter, December 2019 to February 2020, were highly unusual in relation to differences between equivalent periods over the preceding seven years. We also determined if any highly unusual changes persisted into the period July to September 2020.

Methods

The study was undertaken under approval from the Health Research Authority and Health and Care Research Wales, and with the agreement of all NHS neonatal units in England and Wales. Contributing neonatal units and their clinical leads are listed in Supplementary table S1.

For peer review only - http://bmjopen.bmj.com/site/about/guidelines.xhtml

Data sources

Neonatal admissions: We examined the entire population of babies admitted to National Health Service (NHS) neonatal units in England and Wales over the period December 2012 to September 2020. We obtained information on admissions, including the numbers of suspected and proven SARS-CoV-2 cases for mothers and babies, over the study period, from the National Neonatal Research Database (NNRD). This is a national information asset containing detailed clinical information extracted from the electronic patient records of all admissions to NHS neonatal units (16). Data are quality-assured to a research standard (17). As the care of preterm and sick neonates outside of NHS neonatal units is exceptionally rare in the UK, the data comprise the complete population of eligible infants. Neonatal care in England and Wales is delivered in a networked operational model, with babies transferred to higher or lower designation neonatal units according to care needs. Data management procedures for the NNRD therefore include linking episodes of care across neonatal units to provide a complete, single, record from admission to discharge for each baby. No additional data management procedures were undertaken for this study.

Total live and stillbirths: We obtained data on stillbirths and total livebirths from the UK Office for National Statistics (18). The UK definition of stillbirth is when a baby is born dead after 24 completed weeks of pregnancy. A live birth is any baby born with signs of life, regardless of gestational age. If the baby dies before 24 completed weeks, it is called a miscarriage.

Outcomes

We categorised admissions by gestational age as defined by the World Health Organisation (extremely preterm GA1: $<28^{+0}$; very preterm GA2: 28^{+0} to 31^{+6}; moderate to late preterm GA3: 32^{+0} to 36^{+6}; and full term GA4: $\geq 37^{+0}$ weeks^{+days}), ethnicity, using collapsed NHS codes (Asian; Black; White; Mixed/Other) (19), and Index of Multiple Deprivation (IMD) quintile through mapping of the maternal Lower-layer Super Output Area (LSOA) (20). The IMD is the official measure of relative deprivation for small areas in England, formed by combining information from seven weighted domains (income; employment; education, skills and training; health and disability; crime; housing and services; living environment) to produce an overall measure of deprivation. The LSOA defines an area of similar population size, with an average of approximately 1,500 residents or 650 households.

In addition to admissions, we evaluated a range of care processes and key neonatal outcomes. These were: postnatal transfers (downward, from a higher to lower designation neonatal unit; horizontal, to an equivalent designation neonatal unit; upward, from a lower to higher designation neonatal unit); mode of delivery (elective Caesarean section; emergency Caesarean section); all-cause mortality (early neonatal (days 1-7); late neonatal (days 8-28)); intubation at resuscitation, surfactant administration, ligation of patent ductus arteriosus, bronchopulmonary dysplasia (defined as any respiratory support or supplemental oxygen at 36 weeks postmenstrual age), death from or surgery for necrotising enterocolitis, severe brain injury (defined as any seizures, hypoxic ischaemic encephalopathy, intracranial haemorrhage, white matter injury, stroke, central nervous system infection or kernicterus), therapeutic hypothermia, and breast-feeding at discharge.

Analyses

For peer review only - http://bmjopen.bmj.com/site/about/guidelines.xhtml

We compared admissions, processes and outcomes for the initial COVID-19 period April-June 2020 (spring) with the preceding period December 2019-February 2020 (winter) (i.e. spring minus winter difference), and contrasted these differences with the differences for the corresponding pairs of periods in the preceding years from 2013 (i.e. seven sets of paired differences). We made an a priori decision to exclude March 2020, as this represented a period of variable response to the pandemic. We also considered whether any changes between winter and spring 2019-2020 were sustained into July-September 2020 (summer). We did not utilise data prior to 2013 as complete data for England and Wales were not available. We excluded ethnicity from the analysis for Wales, as these data were not available for 2020. We evaluated differences in absolute numbers as well as differences in rates.

We defined the change in each measure during the initial COVID period, April-June 2020 (spring), as "highly unusual" if the difference with the period December 2019-February 2020 (winter) was smaller or larger than all previous corresponding differences. We adopted an empirical Bayes approach to provide a post hoc measure of confidence, or relative strength in the estimate of the difference in rates (21). For each measure and gestational age category, we held out the two three-month periods for the COVID difference (i.e. the spring (Apr 2020-Jun 2020) and winter (Dec 2019-Feb 2020) periods). We then used the 14 corresponding pre-COVID spring and winter three-month periods to estimate the seven background spring-winter differences against which to assess the COVID spring-winter difference. For the 14 pre-COVID three-month periods we identified posterior distributions over the binomial probabilities, approximating them with Gaussian distributions by moment matching and applying shrinkage assuming the individual three-month rates are drawn from a common distribution. We then drew 10,000 independent samples from the fourteen posterior distributions to yield a posterior distribution for each of the seven spring-winter differences. For the seven sets of 10,000 posterior samples we evaluated the proportion that did not meet our criterion for "highly unusual". This provides an estimate of the probability (the p-value) that the COVID period was not "highly unusual". We used a 0.05 threshold as a measure of the strength of the evidence for this conclusion.

We present results in tables and figures showing the periods December-February, April-June and July-September by year, highlighting any highly unusual changes.

Patient and public involvement

The National Neonatal Research Database has been developed in collaboration with parents and former patients; it is overseen by a Steering Board that includes parent representatives. There was no additional patient or public involvement in this specific study.

Results

There were 729,363 admissions to neonatal units in England and Wales over the period December 2012 to September 2020. We identified marked fluctuations in all measures over the eight years. However, during the COVID period April-June 2020, in comparison with the preceding period December-February, there were several changes that were both highly unusual and met our strength of evidence threshold (Table 1). Admissions fell (COVID period difference: total -1492; previous seven-year difference range: +100, +1617; p<0.001; full-term: -1142; +104, +1178; p<0.001; preterm: -350; -26, +477; p<0.001). The absolute number of admissions in all preterm gestational age categories over April-June 2020 (7882) was also

For peer review only - http://bmjopen.bmj.com/site/about/guidelines.xhtml

the lowest for any April-June or December-February period over the previous seven years (range 8505, 9184). The fall in GA1 (extremely preterm) and GA2 (very preterm) admissions, the most immature babies, continued into the period July to September 2020, unlike GA3 (moderate-to-late preterm) and GA4 (full-term) which rose again (Fig 1).

There were highly unusual spring-winter falls in GA1 (extremely preterm) admissions in IMD quintile 1, and GA2 (very preterm) admissions in IMD quintiles 1 and 2, though only the latter had a p-value below 0.05 (-41; -20, +59; p=0.036). There were highly unusual falls in GA4 (full-term) admissions in IMD quintiles 3, 4 and 5, and additionally in GA3 (moderate-to-late preterm) admissions in IMD 5 (Fig 2). The fall in GA1 (extremely preterm) admissions continued into the period July-September. Full-term Black ethnicity admissions rose (+66; -64, +35; p<0.001) in spring, and then fell in the summer (Fig 3), in contrast to spring reductions in total Asian (-137; -14, +101; p<0.001) and total White (-319; -235, +643: p<0.001) groups (Table 1). Transfers to higher designation neonatal units increased (+129; -4, +88; p<0.001). Transfers to lower designation neonatal units decreased (-47; -25, +12; p<0.001).

There were other highly unusual changes. There was a decrease in the number of GA2 (very preterm) babies born by elective Caesarean section (-27; -17, +34; p=0.035). The number of GA1 (extremely preterm) babies born in a hospital with a level 3 (neonatal intensive care) unit fell (-40; +3, +71; p=0.027). The percentage of GA2 (very preterm) babies having surgery for necrotising enterocolitis fell (-1.1%; -0.9%, +0.1%; p=0.017). Breast-feeding at discharge fell in GA3 (moderate-to-late preterm) babies (-202; -91, +170; p=0.031; -1.7%; -1.1%, +1.5%; p=0.047), but rose in GA4 (full-term) babies (+1.4%; -1.2%, +1.0%; p=0.031).

There were also changes that fulfilled our criteria for “highly unusual” but did not meet our strength of evidence threshold, and where numbers were small or where a similar sized effect had occurred during the preceding seven years, casting uncertainty on their relevance. The number of GA4 (full-term) babies born by emergency Caesarean section fell (-186; +45, +500); the percentage requiring intubation at resuscitation rose (+ 0.3%; -0.5, +0.15) as did the proportion with severe brain injury (+0.3%; -0.2, +0.3). The percentage of GA1 (extremely preterm) babies receiving surfactant (+2.5%; -1.6, +1.2) and the number and percentage of GA2 (very preterm) babies receiving surgery for patent ductus arteriosus (N: +2; -5, +1; %: +0.2%; -0.4, +0.1) rose. The percentage of GA3 (moderate-to-late preterm) babies developing bronchopulmonary dysplasia fell (+0.6%; -0.7, +0.1). We identified no highly unusual changes in antenatal steroid use, horizontal transfers, therapeutic hypothermia or early and late neonatal mortality. All outcomes evaluated are shown in the Supplementary Table S2.

We show the number of suspected and confirmed cases of COVID-19 in mothers and babies over the periods December 2019-February 2020, April 2020-June 2020 and July-September 2020 in Table 2. Using Office for National Statistics data, we show changes in stillbirths and livebirths for England and Wales over the study period; these do not suggest a highly unusual change occurred over April-June 2020 (Fig 4).

Discussion

We identified highly unusual changes in key perinatal measures during the immediate period of the first national UK lockdown, although the number of confirmed cases of COVID-19 in babies admitted to neonatal units, and their mothers, was small. Our study included all admissions to NHS neonatal units in England and Wales over an eight-year period. We assessed all preterm and full-term admissions as well as extremely preterm, very preterm, and moderate-to-late preterm groups individually, as degree of immaturity has a cardinal

For peer review only - http://bmjopen.bmj.com/site/about/guidelines.xhtml

influence upon care pathways and morbidities. In view of known seasonal fluctuations in births, we assessed the difference between the immediate period of national COVID-19 lockdown with the preceding quarter, excluding a priori the entire month of March 2020, and compared them with differences in the corresponding epochs of the previous seven years.

We found a highly unusual fall in full-term admissions during the immediate COVID-19 period. This was not due to a fall in total births, or a reduction in elective Caesarean sections, following which infants are more likely to require neonatal unit admission than those born vaginally (15). This suggests a rise in the clinical threshold for the admission of mature babies to neonatal units occurred during the immediate COVID-19 lockdown. Despite the fall in admissions, there was a highly unusual increase in transfers of moderate-to-late preterm and full-term babies to a higher designation neonatal unit. Upward transfer of mature babies is usually only undertaken if higher intensity care is required, suggesting the number with serious illness increased substantially. In this context, the increase in the proportion of full-term babies born by emergency Caesarean section, requiring intubation for resuscitation, and with severe brain injury, should be noted. These changes fulfilled our criteria for highly unusual, although numbers were small and our strength of evidence threshold was not reached. A further notable finding was that the fall in full-term admissions masked a highly unusual increase in the number of admissions of full-term babies of Black ethnicity, contrasting with a decrease in Asian and White ethnic groups. Taken together, our data indicate greater likelihood of late presentation and delayed delivery of mature babies in fetal distress, in accord with the known marked reduction in all healthcare-seeking behaviours with the onset of the pandemic (22, 23), and greater adverse impact upon Black communities (24, 25).

We found evidence of other perturbations to neonatal care pathways. It is a UK standard of care to deliver extremely preterm infants in a hospital with a level 3 (neonatal intensive care) neonatal unit (26). However, during the immediate COVID period there was a highly unusual decrease in the number of extremely preterm babies born in hospitals with a level 3 neonatal unit. This indicates that obstetric *in utero* transfers (transfers of mothers at risk of extremely preterm delivery to a tertiary centre) were less likely. The fall in total admissions meant it was important to evaluate the proportion of babies experiencing a particular outcome. We identified changes that though fulfilling our criteria for highly unusual, and meeting our strength of evidence threshold, were small, and may have occurred by chance. These included a decrease in the proportion of very preterm babies receiving surgery for necrotising enterocolitis and an increase in the proportion of full-term babies breast-feeding at discharge.

We also identified a highly unusual fall in all preterm admissions, though we were unable to distinguish between spontaneous and medically indicated preterm births. The numbers of moderate-to-late preterm babies dominate the preterm category, and a fall in their admission numbers may, as with full-term babies, reflect a rise in clinical thresholds. However, we also found a highly unusual fall in extremely preterm admissions, those born below 28 weeks gestation, a change that appeared confined to the two lowest IMD quintiles representing the most deprived groups. In both, the fall continued into the period July-September 2020. The absolute numbers of extremely preterm babies, even in a whole population dataset, are small, hence it is unsurprising that even though highly unusual, the fall did not meet our stringent statistical threshold. There have however been seven previous reports of a fall in preterm births during the immediate COVID-19 period, though all involved substantially smaller numbers than our study (6-12). Berghella et al compared records from a single

For peer review only - http://bmjopen.bmj.com/site/about/guidelines.xhtml

hospital in northeast United States over March 1 to July 31 2020, with the same period in 2019 (6). They identified seven births below 28 weeks gestation in 2020, compared with fourteen in the previous year. Philip et al compared births at a regional hospital in Ireland over Jan 1-April 30, 2020 with the same period of the preceding nineteen years, identifying only three very and extremely low birthweight infants compared to a predicted number of eight (7). However, Ireland implemented lockdown measures in early March, not in early January, weakening the inference of a temporal association. Been et al used a difference-in-regression-discontinuity approach to study the impact on preterm births of COVID-19 mitigation measures introduced at three points in March 2020 in the Netherlands. They identified a statistically significant reduction only in moderate-to-late preterm births and only in relation to the first time-point (8). Hedermann et al compared the period March 12 to April 14, 2020 with the average rate in Denmark over the previous five years (9). They identified only fifty-eight extremely preterm births over the five-year period and noted extremely preterm births were significantly lower in 2020, but not very or moderate-to-late preterm births. They were unable to exclude the possibility of a corresponding rise in late abortions or stillbirths. Matheson et al studied births in three maternity hospitals in Melbourne, Australia, identifying nine extremely preterm births over July-September 2020, compared with twenty during the same period in 2019 (10). Lemon et al describe a decrease in preterm births in a single US hospital limited to White women from more advantaged neighbourhoods (11). Maeda et al studied records from 186 Japanese acute care hospitals noting a decrease in preterm births but the 95% confidence interval for the adjusted incidence rate ratios included or were close to one (below 34 weeks gestation: 0.71; 95% CI, 0.50 to 1.00; below 37 weeks: 0.85; 95% CI, 0.74 to 0.98) and the extent of population coverage is not known (12). Handley et al noted no decrease in preterm births in two Philadelphia hospitals (13). Comparisons between the UK and US are problematic, first because the healthcare systems are very different, and second, because US reports are centre rather than population-based, and hence at risk of ascertainment bias.

All these studies compared a COVID period with earlier periods. In such a direct comparison there is an implicit assumption that COVID-19 is the only agent likely to have influenced the outcome. However, as we show, there have been marked fluctuations in outcomes over previous years. As the onset and duration of other influences is unknown, subsuming them into the residual error term of a model risks deriving a flawed estimate. In contrast to these studies, we considered the *differences* between three-month pre- and post-COVID periods and compared them to the corresponding three-month differences over seven previous years. By comparing differences we are able to assess the strength of a change during the COVID period taking other, unknown, influences into account. We acknowledge, however, that a limitation of our approach is that our measure of exceptionality may be too conservative, potentially hindering detection of a COVID-19 effect. We also acknowledge that we made no adjustment for multiple comparisons as our p-values were used solely for evaluating the relative strength of evidence. Our approach is aligned with other Bayesian approaches (27) and our exploration of population-based data should be regarded more as a hypothesis generating rather than a hypothesis testing analysis.

We identified a fall in extremely preterm admissions over April-June 2020 in comparison to December 2019-February 2020, whereas in all previous seven years the number rose over corresponding periods. In the UK, all extremely preterm babies are admitted to an NHS neonatal unit, hence the fall likely reflects a genuine reduction in live births in this gestational

For peer review only - http://bmjopen.bmj.com/site/about/guidelines.xhtml

age group. Though a small study from a single London hospital, employing a before and after approach, suggested stillbirths rose during the immediate COVID period (28), this is not supported by data from the Office for National Statistics. Our finding that the highly usual reduction in extremely preterm admissions during the immediate COVID national lockdown occurred in the most deprived socio-economic groups and was sustained into the following three months, is intriguing. Globally, preterm birth rates are increasing, with a strong association with poverty, disadvantage and deprivation (29). Attempts to lower the preterm birth rate have remained stubbornly resistant to a range of medical interventions over the years, from widespread use of tocolytics, bedrest, cervical cerclage, vaginal progesterone, and enhanced surveillance. Thus the possibility that non-healthcare related interventions may be effective is important.

In conclusion, our observation of a fall in extremely preterm admissions during the immediate period of national COVID-19 lockdown, sustained in lower socio-economic groups into the subsequent three months, requires corroboration, and we hope data will be forthcoming from other large, population-based birth cohorts. Our findings should also provide impetus to study the effects on preterm births of public health interventions, such as improved air quality, reduced exposure to crowded environments, altered working during the second trimester of pregnancy, and their interactions with other trigger events, and with socio-economic status and ethnicity. The reasons for the fall in admissions of more mature babies are more likely to be related to changes in clinical thresholds. Together with evidence of perturbations in care pathways, these findings justify consideration of preparedness and public messaging during national crises adding weight to calls for an official COVID-19 inquiry into UK Government actions (30), such as the recommendation to rely upon the call service NHS111 for medical advice (31), that has now been agreed but deferred until the spring of 2022 (32). Finally, the highly unusual rise in admissions of full-term Black ethnicity babies, contrasted with a fall in all other ethnic groups, adds to the growing evidence of a disproportionately higher adverse impact upon this demographic group and speaks to the moral imperative to address ethnic and socio-economic health disparities urgently, as well as growing calls for investment in research to improve maternal and newborn health (33).

Acknowledgements

We gratefully acknowledge the contribution of all UK Neonatal Collaborative neonatal units to the National Neonatal Research Database, Mr Richard Colquhoun, Research Manager and data analysts Victor Banda and Julia Lanoue.

Contributor statement

For peer review only - http://bmjopen.bmj.com/site/about/guidelines.xhtml

All authors had full access to all study data and take responsibility for the integrity of the data, the accuracy of the analysis, and the decision to submit for publication. The study was conceived by NM, CB and SU; data were prepared by KO and SG; the analysis was conducted by NL, EDA and SG; figures were prepared by NL; the paper was written by NM; all authors reviewed and contributed to the final draft submitted; the guarantor is NM.

Transparency declaration

The lead author NM affirms that the manuscript is an honest, accurate, and transparent account of the study being reported; that no important aspects of the study have been omitted.

The Corresponding Author has the right to grant on behalf of all authors and does grant on behalf of all authors, an exclusive licence on a worldwide basis to the BMJ Publishing Group Ltd to permit this article to be published in BMJ editions and any other BMJPGL products and sub-licences such use and exploit all subsidiary rights, as set out in the BMJ licence.

Funding source

We acknowledge funding for SFG through a Medical Research Council award (MR/T016752/1) and for NL through an award from the Health Data Research UK Hub Discover-NOW (reference not applicable), both held by NM. The funders had no role in study design, collection, analysis, and interpretation of data, writing of the report, or the decision to submit the paper for publication.

Competing interest statement

All authors have completed the Unified Competing Interest form and declare no support from any organisation for the submitted work, no financial relationships with any organisations that might have an interest in the submitted work in the previous three years, no other relationships or activities that could appear to have influenced the submitted work. NM reports grants outside the submitted work from the Medical Research Council, National Institute of Health Research, March of Dimes, British Heart Foundation, HCA International, Health Data Research UK, Shire Pharmaceuticals, Chiesi Pharmaceuticals, Prolacta Life Sciences, and Westminster Children's Research Fund; NM is a member of the Nestle Scientific Advisory Board and accepts no personal remuneration for this role. NM reports travel and accommodation reimbursements from Chiesi, Nestle and Shire. NM is the Chief Investigator for the National Neonatal Research Database. All other authors report no declarations of interest.

Data sharing statement

The National Neonatal Research Database is a UK national data asset. Details of access procedures are available https://www.imperial.ac.uk/neonatal-data-analysis-unit/neonatal-data-analysis-unit/utilising-the-national-neonatal-research-database/

Ethics Statement

This study was conducted under approval by the UK Research Ethics Service (London Queen Square Research Ethics Committee 21/LO/0024)

References

For peer review only - http://bmjopen.bmj.com/site/about/guidelines.xhtml

1 https://www.instituteforgovernment.org.uk/sites/default/files/timeline-lockdown-web.pdf [Last accessed 04-05-21]

2 Jephcote C, Hansell AL, Adams K, Gulliver J. Changes in air quality during COVID-19 'lockdown' in the United Kingdom Environmental Pollution 2021; 272:116011

3 UK Office for National Statistics. Domestic abuse during the coronavirus (COVID-19) pandemic, England and Wales: November 2020 https://www.ons.gov.uk/peoplepopulationandcommunity/crimeandjustice/articles/domesticabuseduringthecoronaviruscovid19pandemicenglandandwales/november2020#main-points (last accessed 27-04-21)

4 Sidpra J, Abomeli D, Hameed B, Baker J, Mankad K. Rise in the incidence of abusive head trauma during the COVID-19 pandemic Arch Dis Child 2020; 106:e14

5 NHS England Feb 18th 2020 https://www.england.nhs.uk/coronavirus/wp-content/uploads/sites/52/2020/03/coronavirus-primary-care-briefing.pdf; last accessed 20-05-21

6 Berghella V, Boelig R, Roman A, Burd J, Anderson K Decreased incidence of preterm birth during coronavirus disease 2019 pandemic (Research Letter). Am J Obstet Gynecol MFM 2020; 2:100258

7 Philip RK, Purtill H, Reidy E et al. Unprecedented reduction in births of very low birthweight (VLBW) and extremely low birthweight (ELBW) infants during the COVID-19 lockdown in Ireland: a 'natural experiment' allowing analysis of data from the prior two decades. BMJ Glob Health. 2020; 5(9):e003075

8 Been JV, Burgos Ochoa L, Bertens LCM, Schoenmakers S, Steegers EAP, Reiss IKM. Impact of COVID-19 mitigation measures on the incidence of preterm birth: a national quasi-experimental study. Lancet Public Health 2020; 5: e604–11

9 Hedermann G, Hedley PL, Baekvad-Hansen M, et al. Danish premature birth rates during the COVID-19 lockdown Arch Dis Child Fetal Neonatal Ed (2020) published online Aug 9; https://doi.org/10.1136/archdischild-2020-319990

10 Matheson A, McGannon CJ, Malhotra A et al. Prematurity rates during the coronavirus disease 2019 (COVID-19) pandemic lockdown in Melbourne, Australia. Obstet Gynecol 2021; 137:405-7

11 Lemon L, Edwards RP, Simhan HN. What is driving the decreased incidence of preterm birth during the coronavirus disease 2019 pandemic? Am J Obstet Gynecol MFM 2021; 3:100330

12 Maeda Y, Nakamura M, Ninomiya H, Ogawa K, Sago H, Miyawak A. Trends in intensive neonatal care during the COVID-19 outbreak in Japan. Arch Dis Child Fetal Neonatal Ed 2021; 106:F327-9

13 Handley SC, Mullin AM, Elovitz MA et al. Changes in preterm birth phenotypes and stillbirth at 2 Philadelphia hospitals during the SARS-CoV-2 pandemic, March-June 2020 (Research Letter). JAMA 2021; 325(1):87-9

14 Goldenberg RL, Culhane JF, Iams JD, Romero R. Epidemiology and causes of preterm birth Lancet 2008; 371:75-84

For peer review only - http://bmjopen.bmj.com/site/about/guidelines.xhtml

15 Morrison JJ, Rennie JM, Milton PJ. Neonatal respiratory morbidity and mode of delivery at term: influence of timing of elective caesarean section. Br J Obstet Gynaecol 1995; 102:101-6

16 Modi N, Ashby D, Battersby C et al. Developing routinely recorded clinical data from electronic patient records as a national resource to improve neonatal health care: the Medicines for Neonates research programme NIHR Journals Library Southampton (UK) 2019 Sep

17 Battersby C, Statnikov Y, Santhakumaran S et al. The United Kingdom National Neonatal Research Database: A validation study. PloS one. 2018;13(8)

18 Office for National Statistics https://www.ons.gov.uk/peoplepopulationandcommunity/birthsdeathsandmarriages/livebirths/articles/provisionalbirthsinenglandandwales/2020; (Last accessed 04-05-21)

19 NHS Data Model and Dictionary https://datadictionary.nhs.uk/data_elements/pds_ethnic_category_code.html; (Last accessed 04-05-21)

20 Ministry of Housing, Communities and Local Government. The English Indices of Deprivation 2019 https://assets.publishing.service.gov.uk/government/uploads/system/uploads/attachment_data/file/853811/IoD2019_FAQ_v4.pdf; (Last accessed 04-05-21)

21 Casella G. An Introduction to Empirical Bayes Data Analysis. The American Statistician, May 1985, Vol. 39, No. 2 (May, 1985), pp. 83-87

22 Charlesworth A. Shock to the system: COVID-19's long-term impact on the NHS. The Health Foundation 28th May 2020; https://www.health.org.uk/news-and-comment/blogs/shock-to-the-system-covid-19s-long-term-impact-on-the-nhs (last accessed 27-04-21)

23 Morello F, Bima P, Ferreri E et al. After the first wave and beyond lockdown: long-lasting changes in emergency department visit number, characteristics, diagnoses, and hospital admissions Intern Emerg Med 2021;March 8:1-8 Online ahead of print

24 Kirby T Evidence mounts on the disproportionate effect of COVID-19 on ethnic minorities. Lancet Respiratory Medicine 2020; 8:548

25 Public Health England Beyond the data: Understanding the impact of COVID-19 on BAME groups https://assets.publishing.service.gov.uk/government/uploads/system/uploads/attachment_data/file/892376/COVID_stakeholder_engagement_synthesis_beyond_the_data.pdf (Last accessed 04-05-21)

26 British Association of Perinatal Medicine 2019. Perinatal management of extremely preterm birth https://www.bapm.org/resources/80-perinatal-management-of-extreme-preterm-birth-before-27-weeks-of-gestation-2019; (Last accessed 04-05-21)

27 Sjölander A, Vansteelandt S Frequentist versus Bayesian approaches to multiple testing. European Journal of Epidemiology 2019; 34:809-21

For peer review only - http://bmjopen.bmj.com/site/about/guidelines.xhtml

28 Khalil A, von Dadelszen P, Draycott T, Ugwumadu A, O'Brien P, Magee L. Change in the incidence of stillbirth and preterm delivery during the COVID-19 pandemic; Research Letter. JAMA. 2020; 324:705-6

29 Weightman AL, Morgan HE, Shepherd MA, Kitcher H, Roberts C, Dunstan FD. Social inequality and infant health in the UK: systematic review and meta-analyses. BMJ Open 2012 Jun 14; 2(3):e000964

30 Goodman J, de Prudhoe K, Williams C of the Covid-19 Bereaved Families for Justice. UK COVID-19 public inquiry needed to learn lessons and save lives. The Lancet 2021; 397: 177-80

31 https://www.theguardian.com/society/2021/mar/25/patient-safety-watchdog-launches-investigation-into-nhs-111-advice-to-covid-patients; last accessed 20-05-21

32 https://www.gov.uk/government/speeches/pm-house-of-commons-statement-on-covid-12-may-2021; last accessed 14-05-21

33 Modi N, Hanson M Health of women and children is central to covid-19 recovery BMJ 2021; 373:n899

For peer review only

For peer review only - http://bmjopen.bmj.com/site/about/guidelines.xhtml

Table 1

Summary of *highly unusual* changes in admissions to neonatal units in England and Wales during April-June 2020 (spring), the first three months of national COVID-19 lockdown

We considered a change *highly unusual* if the difference (whether positive or negative) between this period and December 2019-February 2020 (winter) was greater than the corresponding differences for all seven preceding years, or was in the opposite direction to all previous differences regardless of magnitude

The P value reflects the uncertainty in the comparison of the spring-winter 2019-2020 differences and spring-winter differences in the previous seven years; the table lists all results for which the P value is less than 0.05

N=absolute number; %=percentage of infants admitted in gestational age category

Highly unusual changes	Gestational age category	Direction of change (Apr-Jun 2020 compared with Dec 2019-Feb 2020	Absolute magnitude of change (Apr-Jun 2020 compared with Dec 2019-Feb 2020	Range of change between Apr-Jun and preceding Dec-Feb in the years 2012-2019	P value
Total babies admitted (N)	• All preterm<br>• Full-term<br>• All admissions	Decrease<br>Decrease<br>Decrease	-350<br>-1142<br>-1492	-26, +479<br>+104, +1178<br>+100, +1617	<0.001<br><0.001<br><0.001
Black ethnicity (N)	• Full-term	Increase	+66	-64, +35	<0·001
Asian ethnicity (N)	• All admissions	Decrease	-137	-14, +101	<0.001
White ethnicity (N)	• Full-term<br>• All admissions	Decrease<br>Decrease	-218<br>-319	-21, +365<br>-235, +643	<0.001<br><0.001
Socio-economic quintile two	• Very preterm	Decrease	-41	-20, +59	0.036
Socio-economic quintile three	• Full-term	Decrease	-148	+28, +307	<0.001
Socio-economic quintile four	• Full-term	Decrease	-135	-39, +198	<0.001

For peer review only - http://bmjopen.bmj.com/site/about/guidelines.xhtml

Socio-economic quintile five (least deprived)	• Moderate to late preterm<br>• Full-term	Decrease<br>Decrease	-51<br>-175	-8, +58<br>+17, +164	<0.001<br><0.001
Elective Caesarean section (N)	• Very preterm	Decrease	-27	-17, +34	0.035
Elective Caesarean section (%)	• Very preterm	Decrease	-2.3%	-1.3, +2.0	0.035
Born in hospital with level 3 neonatal unit (intensive care) (N)	• Extremely preterm	Decrease	-40	+3, +71	0.027
Transfer to higher designation neonatal unit (N)	• Moderate-to-late preterm<br>• Full-term<br>• All admissions	Increase<br>Increase<br>Increase	+37<br>+69<br>+129	-8, +18<br>+10, +53<br>-4, +88	0.007<br><0.001<br><0.001
Transfer to lower designation neonatal unit (N)	• Full-term<br>• All admissions	Decrease<br>Decrease	-15<br>-47	-8, +3<br>-25, +12	0.004<br><0.001
Necrotising enterocolitis surgery (%)	• Very preterm	Decrease	-1.1%	-0.9%, +0.1%	0.017
Breast-feeding at discharge (N)	• Moderate-to-late preterm<br>• Full-term	Decrease<br>Decrease	-202<br>-65	-91, +170<br>-38, +267	0.031<br>0.015
Breast-feeding at discharge (%)	• Moderate-to-late preterm<br>• Full-term	Decrease<br>Increase	-1.7%<br>+1.4%	-1.1%, +1.5%<br>-1.2%, +1.0%	0.047<br>0.031

For peer review only - http://bmjopen.bmj.com/site/about/guidelines.xhtml

Table 2 Numbers of mothers and babies with suspected and confirmed SARS-CoV-2 infection

	Mother		Baby	
	Suspected	Confirmed	Suspected	Confirmed
Dec 2019 - Feb 2020	22	9	46	8
Apr 2020 - Jun 2020	486	89	139	13
Jul 2020 - Sep 2020	189	42	20	3

For peer review only

Figure legends

Figure 1

Admissions to neonatal units in England and Wales by gestational age category, and year

GA1: Extremely preterm; GA2: very preterm; GA3: moderate-to-late preterm; GA4: full-term; black circle: December to February; black square: April to June; grey triangle: July to September. The COVID period is highlighted; the thick black lines indicate a change that was highly unusual.

There was a highly unusual fall in all preterm (GA groups 1-3 combined) and full-term (GA4) admissions during the period April to June 2020. The falls in GA1 and GA3 admissions were individually also highly unusual; the falls in GA1 and GA2, the most immature babies, continued into the period July to September 2020, unlike GA3 and GA4 which rose again.

Figure 2

Admissions to neonatal units in England and Wales by gestational age category, year and Index of Multiple Deprivation (IMD) quintile

Black circle: December to February; black square: April to June; grey triangle: July to September. The COVID period is shaded; the thick black lines indicate a change that was highly unusual; GA1: Extremely preterm; GA2: very preterm; GA3: moderate-to-late preterm; GA4: full-term; Q1: quintile 1 (most deprived); Q5: least deprived)

There were highly unusual falls in GA1 (extremely preterm) admissions in IMD quintiles 1 and 2, and in GA2 (very preterm) admissions in IMD quintile 2 over April-June 2020; the fall in GA1 (extremely preterm) admissions was sustained into the period July to September. In contrast, there was a highly unusual fall in GA 3 (moderate-to-late preterm) admissions over the COVID period only in IMD quintile 5 and in GA4 (full-term) admissions in quintiles 2, 3, 4 and 5.

Figure 3

Admissions of Black ethnicity babies to neonatal units in England and Wales by year and period

GA1: Extremely preterm; GA2: very preterm; GA3: moderate-to-late preterm; GA4: full-term; black circle: December to February; black square: April to June; grey triangle: July to September. The COVID period is highlighted; the thick black lines indicate a change that was highly unusual.

There was a highly unusual increase in all admissions (GA groups 1-4 combined) over April to June 2020, driven by the full-term (GA4) category. This increase was not sustained into the period July to September 2020

Figure 4

Live births and stillbirths births, England and Wales by 2013 to 2020 and period

Black circle: December to February; black square: April to June; grey triangle: July to September; The COVID period is highlighted.

For peer review only - http://bmjopen.bmj.com/site/about/guidelines.xhtml

For peer review only

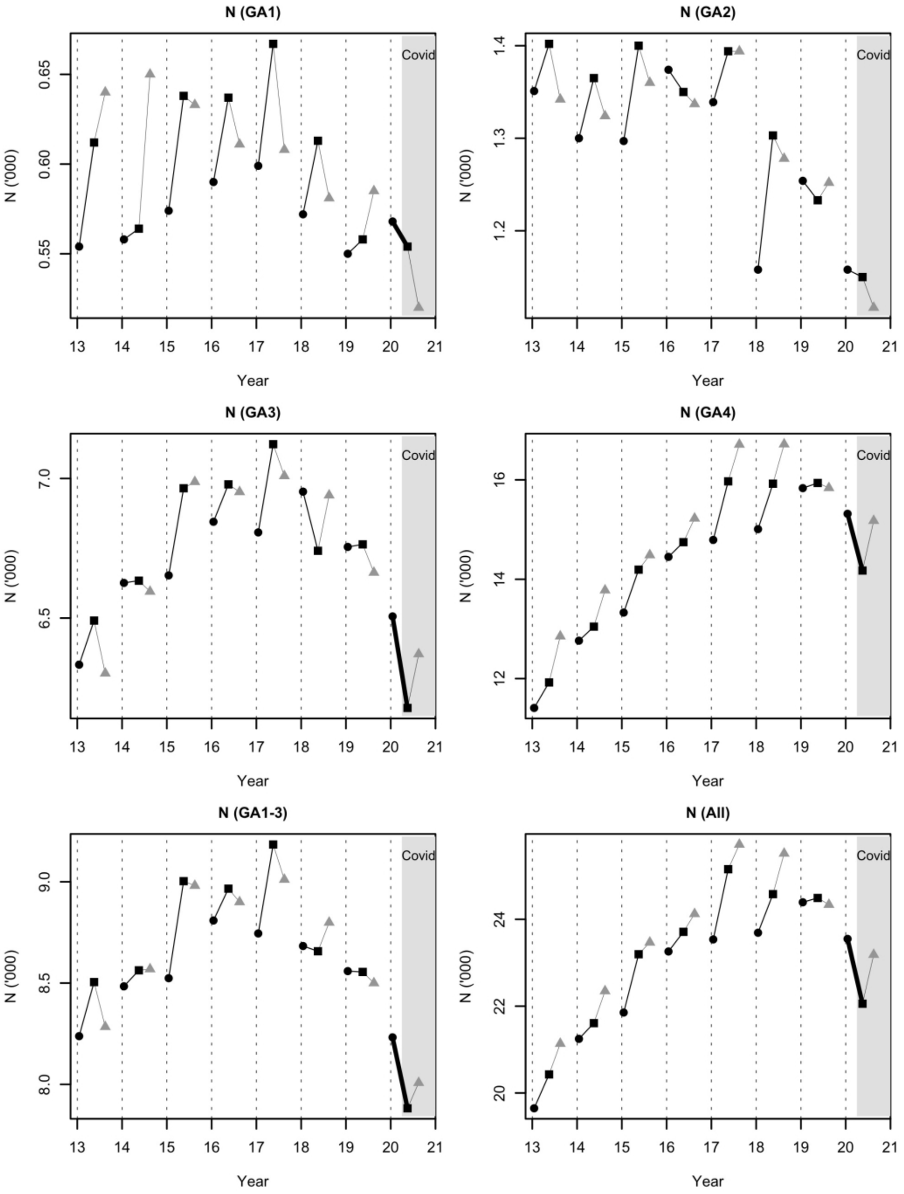

Figure 1
Admissions to neonatal units in England and Wales by gestational age category, and year
GA1: Extremely preterm; GA2: very preterm; GA3: moderate-to-late preterm; GA4: full-term; black circle: December to February; black square: April to June; grey triangle: July to September. The COVID period is highlighted; the thick black lines indicate a change that was highly unusual.
There was a highly unusual fall in all preterm (GA groups 1-3 combined) and full-term (GA4) admissions during the period April to June 2020. The falls in GA1 and GA3 admissions were individually also highly unusual; the falls in GA1 and GA2, the most immature babies, continued into the period July to September 2020, unlike GA3 and GA4 which rose again.

698x920mm (72 x 72 DPI)

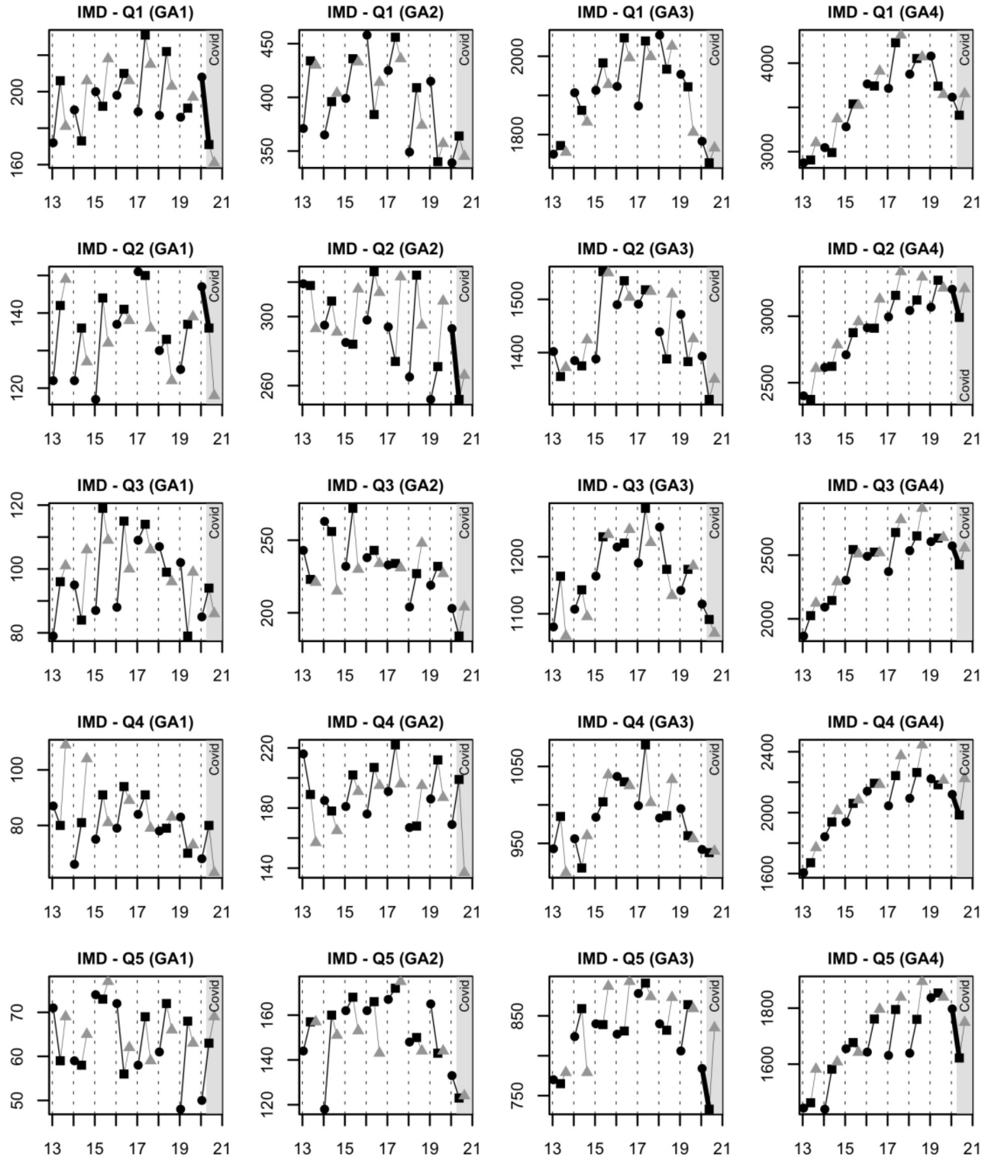

Figure 2

Admissions to neonatal units in England and Wales by gestational age category, year and Index of Multiple Deprivation (IMD) quintile

Black circle: December to February; black square: April to June; grey triangle: July to September. The COVID period is shaded; the thick black lines indicate a change that was highly unusual; GA1: Extremely preterm; GA2: very preterm; GA3: moderate-to-late preterm; GA4: full-term; Q1: quintile 1 (most deprived); Q5: least deprived)

There were highly unusual falls in GA1 (extremely preterm) admissions in IMD quintiles 1 and 2, and in GA2 (very preterm) admissions in IMD quintile 2 over April-June 2020; the fall in GA1 (extremely preterm) admissions was sustained into the period July to September. In contrast, there was a highly unusual fall in GA 3 (moderate-to-late preterm) admissions over the COVID period only in IMD quintile 5 and in GA4 (full-term) admissions in quintiles 2, 3, 4 and 5.

698x825mm (72 x 72 DPI)

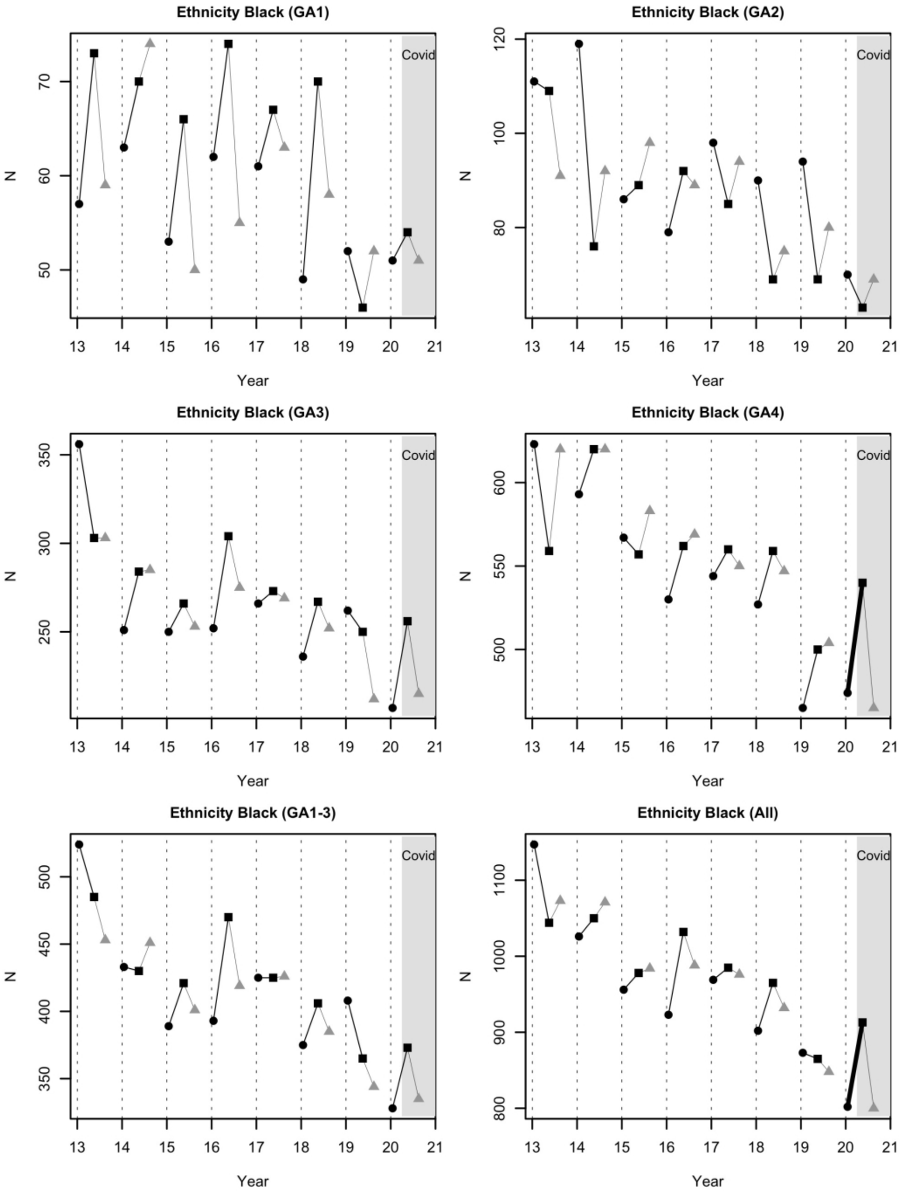

Figure 3
Admissions of Black ethnicity babies to neonatal units in England and Wales by year and period
GA1: Extremely preterm; GA2: very preterm; GA3: moderate-to-late preterm; GA4: full-term; black circle: December to February; black square: April to June; grey triangle: July to September. The COVID period is highlighted; the thick black lines indicate a change that was highly unusual.
There was a highly unusual increase in all admissions (GA groups 1-4 combined) over April to June 2020, driven by the full-term (GA4) category. This increase was not sustained into the period July to September 2020

698x920mm (72 x 72 DPI)

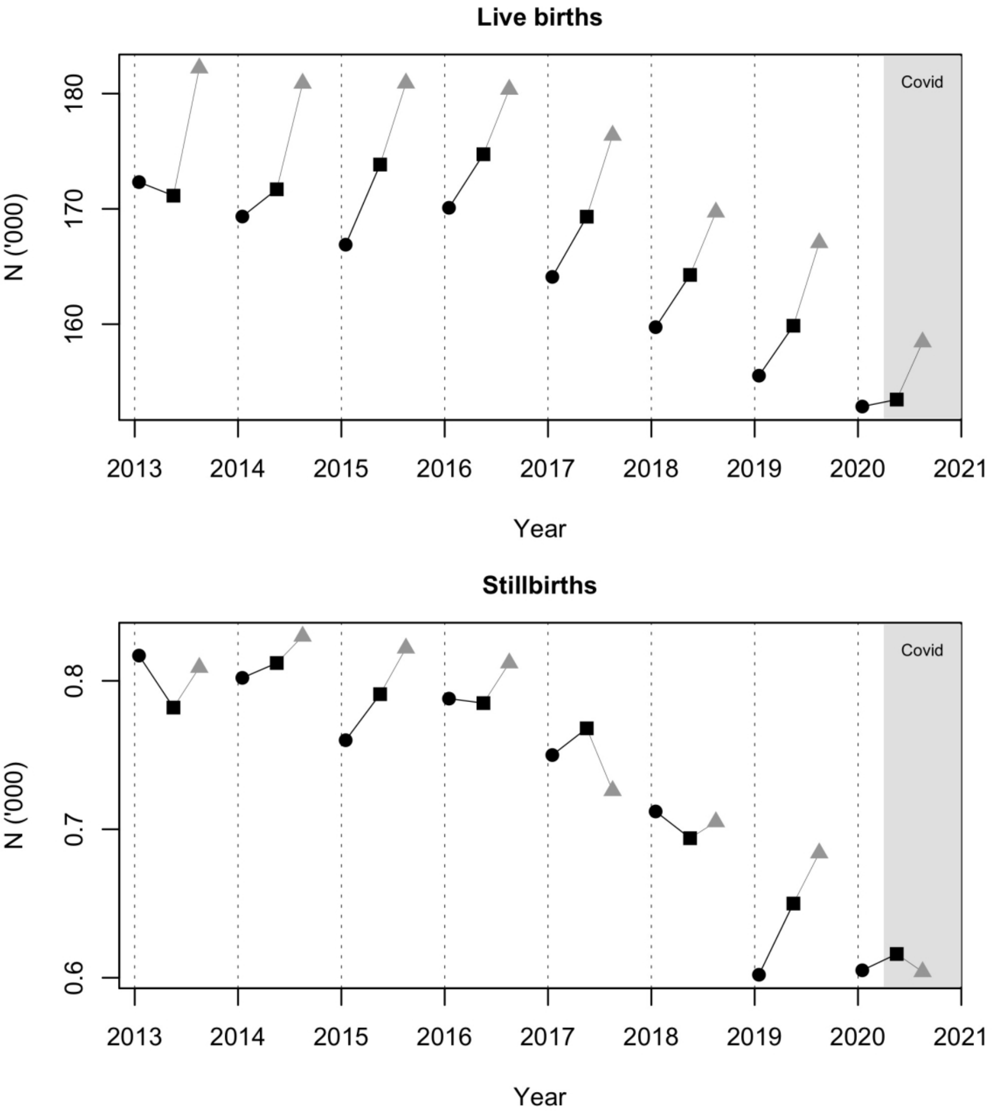

Figure 4
Live births and stillbirths births, England and Wales by 2013 to 2020 and period
Black circle: December to February; black square: April to June; grey triangle: July to September; The COVID period is highlighted.

698x793mm (72 x 72 DPI)

Supplementary Table S1

UK Neonatal Collaborative hospitals and lead clinicians in England and Wales

Hospital	Lead clinician
Airedale General	Dr Matthew Babirecki
Arrowe Park	Dr Anand Kamalanathan
Barnet	Dr Tim Wickham
Barnsley District General	Dr Kavi Aucharaz
Basildon	Dr Aashish Gupta
Basingstoke & North Hampshire	Dr Nicola Paul
Bassetlaw District General	Dr L M Wong
Bedford	Dr Anita Mittal
Birmingham City	Dr Penny Broggio
Birmingham Heartlands	Dr Pinki Surana
Birmingham Women's	Dr Matt Nash
Bradford Royal Infirmary	Dr Sunita Seal
Broomfield, Chelmsford	Dr Ahmed Hassan
Calderdale Royal Hospital	Dr Karin Schwarz
Chelsea & Westminster	Dr Shu-Ling Chuang
Chesterfield & North Derbyshire Royal	Dr Aiwyne Foo
Colchester General	Dr Jo Anderson
Conquest	Dr Graham Whincup
Countess of Chester	Dr Stephen Brearey
Croydon University	Dr John Chang
Cumberland Infirmary	Dr Yee Aung
Darent Valley	Dr Abdul Hasib
Darlington Memorial Hospital	Dr Mehdi Garbash
Derriford Hospital	Dr Alex Allwood
Diana Princess of Wales	Dr Pauline Adiotomre
Doncaster Royal Infirmary	Dr Nigel Brooke
Dorset County	Dr Abby Deketelaere
East Surrey	Dr K Abdul Khader
Epsom General	Dr Ruth Shephard
Frimley Park	Dr Sanghavi Rekha
Furness General	Dr Anas Olabi
George Eliot	Dr Mukta Jain
Gloucester Royal	Dr Jennifer Holman
Good Hope	Dr Pinki Surana
Great Western	Dr Stanley Zengeya
Guy's & St Thomas'	Dr Geraint Lee
Harrogate District	Dr Sobia Balal
Hereford County	Dr Cath Seagrave
Hillingdon	Dr Tristan Bate
Hinchingbrooke	Dr Hilary Dixon
Homerton	Dr Narendra Aladangady
Hull Royal Infirmary	Dr Hassan Gaili
Ipswich	Dr Matthew James
James Cook University	Dr M Lal

For peer review only

James Paget	Dr Ambadkar
Kettering General	Dr Poornima Pandey
Kings College	Dr Ravindra Bhat
King's Mill	Dr Simon Rhodes
Kingston	Dr Vinay Pai
Lancashire Women and Newborn Centre	Dr Savi Sivashankar
Leeds	Dr Lawrence Miall
Leicester General	Dr Jonathan Cusack
Leicester Royal Infirmary	Dr Venkatesh Kairamkonda
Leighton	Dr Michael Grosdenier
Lincoln County	Dr Kollipara
Lister	Dr J Kefas
Liverpool Women's	Dr Christopher Dewhurst
Luton & Dunstable	Dr Jennifer Birch
Macclesfield District General	Dr Gail Whitehead
Manor	Dr Krishnamurthy
Medway Maritime	Dr Ghada Ramadan
Milton Keynes General	Dr I Misra
Musgrove Park	Dr Chris Knight
New Cross	Dr Tilly Pillay
Newham General	Dr Imdad Ali
Nobles	Dr Prakash Thiagarajan
Norfolk & Norwich University	Dr Mark Dyke
North Devon District	Dr Michael Selter
North Manchester General	Dr P Kamath
North Middlesex University	Dr Neeraj Jain
Northumbria Specialist Emergency Care	Vivien Spencer
Northampton General	Dr Subodh Gupta
Northwick Park	Dr Richard Nicholl
Nottingham City	Dr Steven Wardle
Nottingham University	Dr Steven Wardle
Ormskirk District General	Dr Andreea Bontea
John Radcliffe	Dr Eleri Adams
Peterborough City	Dr Katharine McDevitt
Pilgrim	Dr Ajay Reddy
Pinderfields General (Pontefract General Infirmary)	Dr David Gibson
Poole General	Prof Minesh Khashu
Princess Alexandra	Dr Chinnappa Reddy
Princess Anne	Dr Mark Johnson
Princess Royal	Dr P Amess
Princess Royal (previously Royal Shrewsbury)	Dr Deshpande
Princess Royal University	Dr Elizabeth Sleight
Queen Alexandra	Dr Charlotte Groves
Queen Charlotte's	Dr Lidia Tyszczuk
Queen Elizabeth, Gateshead	Dr Dennis Bosman
Queen Elizabeth, King's Lynn	Dr Glynis Rewitzky
Queen Elizabethl, Woolwich	Dr Olutoyin Banjoko
Queen Elizabeth the Queen Mother	Dr Bushra Abdul-Malik

For peer review only

Queen's Hospital, Burton on Trent	Dr Dominic Muogbo
Queen's Hospital, Romford	Dr Khalid Mannan
Queen's Hospital, Romford 2	Dr Anand Shirsalkar
Rosie Maternity, Addenbrookes	Dr Angela D'Amore
Rotherham District General	Dr Shameel Mattara
Royal Albert Edward Infirmary	Dr Christos Zipitis
Royal Berkshire	Dr Peter De Halpert
Royal Bolton	Dr Paul Settle
Royal Cornwall	Dr Paul Munyard
Royal Derby	Dr John McIntyre
Royal Devon & Exeter	Dr David Bartle
Royal Hampshire County	Dr Lucinda Winckworth
Royal Lancaster Infirmary	Dr Joanne Fedee
Royal Oldham	Dr Natasha Maddock
Royal Preston	Dr Richa Gupta
Royal Stoke University	Dr Alison Moore
Royal Surrey County	Dr Ben Obi
Royal Sussex County	Dr Phil Amess
Royal United Hospital	Dr Stephen Jones
Royal Victoria Infirmary	Dr Naveen Athiraman
Russells Hall	Dr Mahadevan
Salisbury District	Dr Jim Baird
Scarborough General	Dr Kirsten Mack
Scunthorpe General	Dr Pauline Adiotomre
Southend	Dr Vineet Gupta
Southmead	Dr Alison Pike
St George's	Dr Charlotte Huddy
St Helier	Dr Salim Yasin
St Mary's, Isle of Wight	Dr Sian Butterworth
St Mary's, London	Dr Lidia Tyszczuk
St Mary's, Manchester	Dr Ngozi Edi-Osagie
St Michael's	Dr Pamela Cairns
St Peter's	Dr Peter Reynolds
St Richard's	Dr Nick Brennan
Stepping Hill	Dr Carrie Heal
Stoke Mandeville	Dr Sanjay Salgia
Sunderland Royal	Dr Majd Abu-Harb
Tameside General	Dr Jacqeline Birch
The Jessop Wing, Sheffield	Dr Porus Bastani
The Royal Free	Dr Marice Theron
The Royal London	Dr Vadivelam Murthy
Torbay	Dr Siba Paul
Tunbridge Wells	Dr Hamudi Kisat
University College	Dr Giles Kendall
University Hospital Coventry	Dr Kate Blake
University Hospital Lewisham	Dr Ozioma Obi
University Hospital of North Durham	Dr Mehdi Garbash
University Hospital of North Tees	Dr Hari Kumar
Victoria Hospital, Blackpool	Dr Chris Rawlingson

For peer review only

Warrington	Dr Delyth Webb
Warwick	Dr Bird
Watford General	Dr Sankara Narayanan
West Cumberland	no lead
West Middlesex University	Dr Eleanor Hulse
West Suffolk	Dr Ian Evans
Wexham Park	Dr Rekha Sanghavi
Whipps Cross University	Dr Caroline Sullivan
Whiston	Dr Ros Garr
Whittington	Dr Wynne Leith
William Harvey	Dr Vimal Vasu
Worcestershire Royal	Dr Liza Harry
Worthing	Dr Katia Vamvakiti
Wythenshawe	Dr Ngozi Edi-Osagie
Yeovil District	Dr Megan Eaton
York District	Dr Sundeep Sandhu

Wales

**Hospital**	**Lead clinician**
Singleton	Dr Arun Ramachandran
Princess of Wales	Dr Kate Creese
Royal Gwent	Dr Sunil Reddy
Nevill Hall Hospital	Dr Sunil Reddy
Glan Clwyd l	Dr Ian Barnard
Wrexham Maelor	Dr Brendan Harrington
Ysbyty Gwynedd	Dr Mike Cronin
University Hospital of Wales	Dr Alok Sharma
Prince Charles	Dr Iyad Al-Muzaffar
Glangwili General	Dr Prem Pitchaikani
Withybush	Dr Vishwa Narayan

For peer review only

Supplementary Table. Changes during the Covid-19 period and the range of the corresponding changes in the previous years (* The direction or the difference is unique in the Covid-19 period.)

Group	Direction	Covid change	Pre-Covid range of changes
		Number of babies (N)	
Extremely preterm	Decrease*	–14	6, 68
Very preterm	—	–8	–24, 145
Moderate-to-late preterm	Decrease	–328	–212, 316
Full term	Decrease*	–1142	104, 1178
		Ethnic groups (N)	
		Asian	
Extremely preterm	—	4	–13, 27
Very preterm	—	–22	–36, 43
Moderate-to-late preterm	—	30	–6, 67
Full term	Decrease	–149	–79, 77
		Black	
Extremely preterm	—	3	–6, 21
Very preterm	—	–7	–43, 13
Moderate-to-late preterm	—	49	–53, 52
Full term	Increase	66	–64, 35
		White	
Extremely preterm	—	4	–22, 58
Very preterm	—	18	–17, 101
Moderate-to-late preterm	—	–123	–239, 182
Full term	Decrease	–218	–21, 365
		Other	
Extremely preterm	Decrease	–9	–5, 10
Very preterm	Decrease	–18	–15, 1
Moderate-to-late preterm	—	–6	–13, 37
Full term	—	–3	–25, 63

For peer review only

Group	Direction	Covid change	Pre-Covid range of changes
	Ethnic groups (%)		
	Asian		
Extremely preterm	—	0.85	–3.75, 4.48
Very preterm	—	–2.07	–2.76, 2.96
Moderate-to-late preterm	—	0.87	–0.40, 1.17
Full term	—	–1.28	–1.28, 0.37
	Black		
Extremely preterm	—	0.65	–1.57, 3.63
Very preterm	—	–0.55	–3.88, 1.51
Moderate-to-late preterm	Increase	1.23	–0.90, 0.83
Full term	Increase	1.03	–0.63, 0.24
	White		
Extremely preterm	—	0.64	–4.92, 3.23
Very preterm	Increase	4.53	–2.37, 4.04
Moderate-to-late preterm	—	–2.01	–2.31, 0.54
Full term	—	0.05	–0.66, 1.23
	Other		
Extremely preterm	Decrease	–2.13	–1.25, 1.15
Very preterm	Decrease	–1.91	–1.14, 0.06
Moderate-to-late preterm	—	–0.09	–0.23, 0.55
Full term	—	0.18	–0.24, 0.59
	CS. emergency (%)		
Extremely preterm	—	0.28	–2.53, 5.80
Very preterm	—	0.53	–5.21, 2.82
Moderate-to-late preterm	Increase	2.05	–1.51, 1.71
Full term	—	–0.12	–0.28, 0.84
	CS. elective (%)		
Extremely preterm	—	0.46	–2.06, 1.30
Very preterm	Decrease	–2.30	–1.27, 1.95
Moderate-to-late preterm	Increase	0.51	–1.06, 0.35
Full term	—	0.99	–0.77, 1.10

For peer review only

Group	Direction	Covid change	Pre-Covid range of changes
	Mortality (%)		
	Died at age 1–7 days		
Extremely preterm	—	–0.72	–0.98, 1.99
Very preterm	—	0.59	–0.36, 0.66
Moderate-to-late preterm	—	0.04	–0.03, 0.24
Full term	—	0.05	0.01, 0.11
	Died at age 8–28 days		
Extremely preterm	—	–0.36	–1.35, 0.40
Very preterm	Decrease	–0.96	–0.12, 0.31
Moderate-to-late preterm	—	0.06	–0.06, 0.10
Full term	—	0.04	–0.03, 0.08
	Transfer (%)		
	Downward		
Extremely preterm	—	0.37	–1.07, 0.60
Very preterm	—	–1.12	–1.22, 1.14
Moderate-to-late preterm	Decrease	–0.30	–0.25, 0.20
Full term	Decrease	–0.09	–0.06, 0.02
	Horizontal		
Extremely preterm	—	0.20	–0.55, 1.00
Very preterm	—	–0.69	–0.79, 0.91
Moderate-to-late preterm	—	–0.12	–0.27, 0.21
Full term	—	0.03	–0.10, 0.19
	Upward		
Extremely preterm	Increase	3.58	–6.14, 2.34
Very preterm	—	0.59	–0.42, 1.16
Moderate-to-late preterm	Increase	0.73	–0.18, 0.23
Full term	Increase	0.67	–0.07, 0.23

For peer review only

Group	Direction	Covid change	Pre-Covid range of changes
		Number of babies (N)	
All preterm	Decrease	–350	–26, 479
Full term	Decrease*	–1142	104, 1178
		Ethnic groups (%)	
		Asian	
All preterm	Increase	3.35	–1.67, 3.25
Full term	Decrease	–3.35	–3.25, 1.67
		Black	
All preterm	—	–0.05	–4.54, 2.96
Full term	—	0.05	–2.96, 4.54
		White	
All preterm	—	0.28	–1.41, 0.48
Full term	—	–0.28	–0.48, 1.41
		Other	
All preterm	—	–2.73	–3.00, 1.77
Full term	—	2.73	–1.77, 3.00
		CS. emergency (%)	
All preterm	—	1.70	–1.48, 1.82
Full term	—	–0.12	–0.28, 0.84
		CS. elective (%)	
All preterm	—	0.02	–0.93, 0.49
Full term	—	0.99	–0.77, 1.10
		Mortality (%)	
		Died at age 1–7 days	
All preterm	—	0.10	–0.09, 0.17
Full term	—	0.05	0.01, 0.11
		Died at age 8–28 days	
All preterm	Decrease	–0.10	–0.09, 0.05
Full term	—	0.04	–0.03, 0.08

For peer review only

Group	Direction	Covid change	Pre-Covid range of changes
Severe brain injury (N)			
Extremely preterm	—	5	–8, 24
Very preterm	Decrease	–1	0, 24
Moderate-to-late preterm	—	8	–23, 20
Full term	—	21	–6, 51
Therapeutic hypothermia (N)			
Extremely preterm	—	2	–2, 4
Very preterm	—	–2	–4, 0
Moderate-to-late preterm	—	1	–8, 13
Full term	—	9	–6, 45
Bronchopulmonary dysplasia (N)			
Extremely preterm	—	3	–12, 38
Very preterm	—	–14	–14, 50
Moderate-to-late preterm	—	12	–32, 25
Full term	Not applicable		
Necrotising enterocolitis (N)			
Extremely preterm	—	–6	–9, 5
Very preterm	Decrease	–13	–10, 2
Moderate-to-late preterm	—	1	–7, 8
Full term	—	0	–3, 0
Antenatal steroids (N)			
Extremely preterm	Decrease*	–14	11, 65
Very preterm	—	–25	–47, 139
Moderate-to-late preterm	—	–178	–230, 269
Full term	Decrease	–46	–20, 72
Intubation at resuscitation (N)			
Extremely preterm	Decrease	–25	–2, 51
Very preterm	—	15	–30, 57
Moderate-to-late preterm	—	–13	–32, 19
Full term	—	20	–41, 46

Group	Direction	Covid change	Pre-Covid range of changes
		Surfactant (N)	
Extremely preterm	—	9	3, 54
Very preterm	—	–1	–39, 62
Moderate-to-late preterm	—	–32	–43, 60
Full term	—	14	–30, 43
		Surgery for patent ductus arteriosus (N)	
Extremely preterm	—	–1	–9, 4
Very preterm	Increase	2	–5, 1
Moderate-to-late preterm	—	0	–1, 2
Full term	—	0	–2, 1
		Born at a level 3 unit (N)	
Extremely preterm	Decrease*	–40	3, 71
Very preterm	—	22	–82, 85
Moderate-to-late preterm	—	–44	–74, 215
Full term	—	–327	–363, 822
		Mother's milk exclusive at discharge (N)	
Extremely preterm	Decrease	–27	–19, 25
Very preterm	—	41	–21, 73
Moderate-to-late preterm	Decrease*	–73	51, 169
Full term	Decrease*	–622	251, 629

For peer review only

Group	Direction	Covid change	Pre-Covid range of changes
		Severe brain injury (%)	
Extremely preterm	—	1.49	–1.78, 1.49
Very preterm	—	–0.05	–0.49, 1.54
Moderate-to-late preterm	—	0.19	–0.41, 0.34
Full term	Increase	0.33	–0.22, 0.31
		Therapeutic hypothermia (%)	
Extremely preterm	—	0.36	–0.41, 0.60
Very preterm	—	–0.17	–0.32, 0.00
Moderate-to-late preterm	—	0.04	–0.14, 0.21
Full term	—	0.20	–0.14, 0.31
		Bronchopulmonary dysplasia (%)	
Extremely preterm	—	2.46	–1.76, 3.14
Very preterm	Decrease	–1.02	–0.97, 3.01
Moderate-to-late preterm	Increase	0.59	–0.72, 0.07
Full term		Not applicable	
		Necrotising enterocolitis (%)	
Extremely preterm	—	–0.88	–2.17, 0.41
Very preterm	Decrease	–1.12	–0.86, 0.10
Moderate-to-late preterm	—	0.02	–0.12, 0.11
Full term	—	0.00	–0.03, 0.00
		Antenatal steroids (%)	
Extremely preterm	—	–0.02	–1.03, 3.74
Very preterm	—	–1.52	–1.92, 1.64
Moderate-to-late preterm	—	–0.08	–1.00, 1.36
Full term	—	–0.02	–0.28, 0.39
		Intubation at resuscitation (%)	
Extremely preterm	—	–2.60	–2.73, 1.21
Very preterm	—	1.46	–3.31, 1.78
Moderate-to-late preterm	—	–0.08	–0.48, 0.37
Full term	Increase	0.29	–0.52, 0.15
		Surfactant (%)	
Extremely preterm	Increase	2.46	–1.57, 1.17
Very preterm	—	–0.49	–2.60, 2.13
Moderate-to-late preterm	—	–0.58	–0.93, 0.95
Full term	—	0.35	–0.25, 0.47

For peer review only

Group	Direction	Covid change	Pre-Covid range of changes
		Surgery for patent ductus arteriosus (%)	
Extremely preterm	—	–0.13	–1.78, 0.67
Very preterm	Increase	0.17	–0.39, 0.07
Moderate-to-late preterm	—	0.00	–0.02, 0.03
Full term	—	0.00	–0.02, 0.01
		Born at a level 3 unit (%)	
Extremely preterm	Decrease	–5.20	–1.36, 5.25
Very preterm	—	2.24	–5.19, 3.00
Moderate-to-late preterm	Increase	1.44	–0.99, 1.22
Full term	—	0.96	–2.60, 1.80
		Mother's milk exclusive at discharge (%)	
Extremely preterm	—	–1.85	–5.00, 1.87
Very preterm	Increase	4.23	–2.92, 3.33
Moderate-to-late preterm	—	0.29	0.00, 2.36
Full term	Decrease	–2.32	–0.14, 3.96

For peer review only

The RECORD statement – checklist of items, extended from the STROBE statement, that should be reported in observational studies using routinely collected health data.

	Item No.	STROBE items	Location in manuscript where items are reported	RECORD items	Location in manuscript where items are reported
**Title and abstract**					
	1	(a) Indicate the study's design with a commonly used term in the title or the abstract (b) Provide in the abstract an informative and balanced summary of what was done and what was found		RECORD 1.1: The type of data used should be specified in the title or abstract. When possible, the name of the databases used should be included.<br><br>RECORD 1.2: If applicable, the geographic region and timeframe within which the study took place should be reported in the title or abstract.<br><br>RECORD 1.3: If linkage between databases was conducted for the study, this should be clearly stated in the title or abstract.	Abstract; page 2<br><br>Abstract; page 2<br><br>Not applicable; no linkage between databases
**Introduction**					
Background rationale	2	Explain the scientific background and rationale for the investigation being reported			Section headed "Introduction"; pages 4-6
Objectives	3	State specific objectives, including any prespecified hypotheses			Section headed "Introduction"; page 4
**Methods**					
Study Design	4	Present key elements of study design early in the paper			Sections headed "Introduction" (page 4)
Setting	5	Describe the setting, locations, and relevant dates, including periods of recruitment, exposure, follow-up, and data collection			Sections headed "Introduction" (page 4), "Methods" (pages

For peer review only

					4); "Analyses" (pages 5-6)
Participants	6	*(a) Cohort study* - Give the eligibility criteria, and the sources and methods of selection of participants. Describe methods of follow-up<br>*Case-control study* - Give the eligibility criteria, and the sources and methods of case ascertainment and control selection. Give the rationale for the choice of cases and controls<br>*Cross-sectional study* - Give the eligibility criteria, and the sources and methods of selection of participants<br><br>*(b) Cohort study* - For matched studies, give matching criteria and number of exposed and unexposed<br>*Case-control study* - For matched studies, give matching criteria and the number of controls per case		RECORD 6.1: The methods of study population selection (such as codes or algorithms used to identify subjects) should be listed in detail. If this is not possible, an explanation should be provided.<br><br>RECORD 6.2: Any validation studies of the codes or algorithms used to select the population should be referenced. If validation was conducted for this study and not published elsewhere, detailed methods and results should be provided.<br><br>RECORD 6.3: If the study involved linkage of databases, consider use of a flow diagram or other graphical display to demonstrate the data linkage process, including the number of individuals with linked data at each stage.	Section headed "Neonatal admissions" (page 5)<br><br>References 16-18<br><br>No linkage of databases
Variables	7	Clearly define all outcomes, exposures, predictors, potential confounders, and effect modifiers. Give diagnostic criteria, if applicable.		RECORD 7.1: A complete list of codes and algorithms used to classify exposures, outcomes, confounders, and effect modifiers should be provided. If these cannot be reported, an explanation should be provided.	Section headed "Outcomes" (page 5)
Data sources/ measurement	8	For each variable of interest, give sources of data and details of methods of assessment (measurement).			Section headed "Data sources" (pages 4-5)

For peer review only

		Describe comparability of assessment methods if there is more than one group			
Bias	9	Describe any efforts to address potential sources of bias			Section headed "Analyses" (pages 5-6)
Study size	10	Explain how the study size was arrived at			Section headed "Neonatal Admissions" (page 5)
Quantitative variables	11	Explain how quantitative variables were handled in the analyses. If applicable, describe which groupings were chosen, and why			Section headed "Outcomes" (page 5)
Statistical methods	12	(a) Describe all statistical methods, including those used to control for confounding<br>(b) Describe any methods used to examine subgroups and interactions<br>(c) Explain how missing data were addressed<br>(d) *Cohort study* - If applicable, explain how loss to follow-up was addressed<br>*Case-control study* - If applicable, explain how matching of cases and controls was addressed<br>*Cross-sectional study* - If applicable, describe analytical methods taking account of sampling strategy<br>(e) Describe any sensitivity analyses			Section headed "Analyses" (pages 5-6)

For peer review only

Data access and cleaning methods		..		RECORD 12.1: Authors should describe the extent to which the investigators had access to the database population used to create the study population.<br><br>RECORD 12.2: Authors should provide information on the data cleaning methods used in the study.	Section headed "Contributor Statement" (page 10)<br><br>Section headed "Neonatal admissions" (page 5)
Linkage		..		RECORD 12.3: State whether the study included person-level, institutional-level, or other data linkage across two or more databases. The methods of linkage and methods of linkage quality evaluation should be provided.	Not applicable
**Results**					
Participants	13	(a) Report the numbers of individuals at each stage of the study (*e.g.*, numbers potentially eligible, examined for eligibility, confirmed eligible, included in the study, completing follow-up, and analysed)<br>(b) Give reasons for non-participation at each stage.<br>(c) Consider use of a flow diagram		RECORD 13.1: Describe in detail the selection of the persons included in the study (*i.e.*, study population selection) including filtering based on data quality, data availability and linkage. The selection of included persons can be described in the text and/or by means of the study flow diagram.	Section headed "Results" (page 6)
Descriptive data	14	(a) Give characteristics of study participants (*e.g.*, demographic, clinical, social) and information on exposures and potential confounders<br>(b) Indicate the number of participants with missing data for each variable of interest			Section headed "Results" (page 6)

For peer review only

		(c) *Cohort study* - summarise follow-up time (*e.g.*, average and total amount)			
Outcome data	15	*Cohort study* - Report numbers of outcome events or summary measures over time<br>*Case-control study* - Report numbers in each exposure category, or summary measures of exposure<br>*Cross-sectional study* - Report numbers of outcome events or summary measures			Section headed "Results" (page 6)
Main results	16	(a) Give unadjusted estimates and, if applicable, confounder-adjusted estimates and their precision (e.g., 95% confidence interval). Make clear which confounders were adjusted for and why they were included<br>(b) Report category boundaries when continuous variables were categorized<br>(c) If relevant, consider translating estimates of relative risk into absolute risk for a meaningful time period			Section headed "Results" (page 6)
Other analyses	17	Report other analyses done—e.g., analyses of subgroups and interactions, and sensitivity analyses			Section headed "Results" (page 6)
**Discussion**					
Key results	18	Summarise key results with reference to study objectives			Section headed "Results" (page 6)
Limitations	19	Discuss limitations of the study, taking into account sources of potential bias or imprecision.		RECORD 19.1: Discuss the implications of using data that were not created or collected to answer the specific research question(s). Include	Paragraph 5 of section headed "Discussion" (page 7)

For peer review only

		Discuss both direction and magnitude of any potential bias		discussion of misclassification bias, unmeasured confounding, missing data, and changing eligibility over time, as they pertain to the study being reported.	
Interpretation	20	Give a cautious overall interpretation of results considering objectives, limitations, multiplicity of analyses, results from similar studies, and other relevant evidence			Sections headed "Discussion" (page 7)
Generalisability	21	Discuss the generalisability (external validity) of the study results			Sections headed "Discussion" (page 7)
**Other Information**					
Funding	22	Give the source of funding and the role of the funders for the present study and, if applicable, for the original study on which the present article is based			Section headed "Funding Source" (page 10)
Accessibility of protocol, raw data, and programming code		..		RECORD 22.1: Authors should provide information on how to access any supplemental information such as the study protocol, raw data, or programming code.	Sections headed "Data Sharing Statement" (page 11)

*Reference: Benchimol EI, Smeeth L, Guttmann A, Harron K, Moher D, Petersen I, Sørensen HT, von Elm E, Langan SM, the RECORD Working Committee. The REporting of studies Conducted using Observational Routinely-collected health Data (RECORD) Statement. *PLoS Medicine* 2015; in press.

*Checklist is protected under Creative Commons Attribution (CC BY) license.

For peer review only
